# Modelling adult *Aedes aegypti* and *Aedes albopictus* survival at different temperatures in laboratory and field settings

**DOI:** 10.1186/1756-3305-6-351

**Published:** 2013-12-12

**Authors:** Oliver J Brady, Michael A Johansson, Carlos A Guerra, Samir Bhatt, Nick Golding, David M Pigott, Hélène Delatte, Marta G Grech, Paul T Leisnham, Rafael Maciel-de-Freitas, Linda M Styer, David L Smith, Thomas W Scott, Peter W Gething, Simon I Hay

**Affiliations:** 1 Spatial Ecology and Epidemiology Group, Department of Zoology, University of Oxford, Tinbergen Building, South Parks Road, Oxford, UK; 2 Dengue Branch, Division of Vector-Borne Diseases, Centers for Disease Control and Prevention, San Juan, Puerto Rico, USA; 3 CIRAD, UMR PVBMT, 97410, Saint Piarre, la Réunion, France; 4 Laboratorio de Investigaciones en Ecología y Sistemática Animal (LIESA), Universidad Nacional de la Patagonia San Juan Bosco, FCN-Sede, Esquel, Chubut, Argentina; 5 Department of Environmental Science & Technology, University of Maryland, College Park, MD 20742, USA; 6 Laboratório de Transmissores de Hematozoários, Instituto Oswaldo Cruz, Fiocruz, Rio de Janeiro, RJ, Brazil; 7 Wadsworth Center, New York State Department of Health, Albany, NY 12208, USA; 8 Department of Epidemiology, Johns Hopkins Bloomberg School of Public Health, Baltimore MD, USA; 9 Department of Entomology, University of California Davis, Davis, CA, USA; 10 Fogarty International Center, National Institutes of Health, Bethesda, MD 20892, USA

**Keywords:** *Aedes*, Survival, Temperature, Mortality, Longevity, Modelling, Mark-release-recapture, Dengue, Transmission, Generalised additive models

## Abstract

**Background:**

The survival of adult female *Aedes* mosquitoes is a critical component of their ability to transmit pathogens such as dengue viruses. One of the principal determinants of *Aedes* survival is temperature, which has been associated with seasonal changes in *Aedes* populations and limits their geographical distribution. The effects of temperature and other sources of mortality have been studied in the field, often via mark-release-recapture experiments, and under controlled conditions in the laboratory. Survival results differ and reconciling predictions between the two settings has been hindered by variable measurements from different experimental protocols, lack of precision in measuring survival of free-ranging mosquitoes, and uncertainty about the role of age-dependent mortality in the field.

**Methods:**

Here we apply generalised additive models to data from 351 published adult *Ae. aegypti* and *Ae. albopictus* survival experiments in the laboratory to create survival models for each species across their range of viable temperatures. These models are then adjusted to estimate survival at different temperatures in the field using data from 59 *Ae. aegypti* and *Ae. albopictus* field survivorship experiments. The uncertainty at each stage of the modelling process is propagated through to provide confidence intervals around our predictions.

**Results:**

Our results indicate that adult *Ae. albopictus* has higher survival than *Ae. aegypti* in the laboratory and field, however, *Ae. aegypti* can tolerate a wider range of temperatures. A full breakdown of survival by age and temperature is given for both species. The differences between laboratory and field models also give insight into the relative contributions to mortality from temperature, other environmental factors, and senescence and over what ranges these factors can be important.

**Conclusions:**

Our results support the importance of producing site-specific mosquito survival estimates. By including fluctuating temperature regimes, our models provide insight into seasonal patterns of *Ae. aegypti* and *Ae. albopictus* population dynamics that may be relevant to seasonal changes in dengue virus transmission. Our models can be integrated with *Aedes* and dengue modelling efforts to guide and evaluate vector control, better map the distribution of disease and produce early warning systems for dengue epidemics.

## Background

Survival of arthropod vectors is one of the most important components of transmission of a vector-borne pathogen [1-3]. Increased survival allows the vector to produce more offspring, to increase the chances of them becoming infected, to disperse over greater distances, to survive long enough to become infectious, and then to deliver more infective bites during the remainder of its lifetime. As a result, small changes in survival rate cause large changes in the rate of pathogen transmission [3-7]. Furthermore, seasonality and the geographic distribution of vector borne diseases are often constrained by differences in vector survival [8,9].

Adult female *Aedes aegypti* (L.) are the principal vectors for dengue and urban yellow fever viruses, two globally important human arboviruses [10-12]. *Ae. albopictus* (Skuse) can also act as a secondary vector for dengue viruses (DENV) [13,14], along with 22 other arboviruses [13,15,16], some of which have an increasing public health burden [17]. There has, therefore, been considerable interest in investigating and quantifying factors affecting survival of adult females of both species and how this contributes to virus transmission [18,19]. Because *Aedes* mosquitoes are small-bodied poikilotherms it is logical that temperature is consistently observed as a principal factor affecting survival [20,21] and the current global range of both species align broadly with separate critical limits imposed by winter isotherms [9,22]. While other factors such as humidity and photoperiod are important, the effects of temperature have been most rigorously quantified and most frequently identified as limiting factors for survival [10,18].

Reconciling the expected difference between laboratory and field survival estimates has been complicated by the lack of precision of available field techniques and the limited temperature ranges over which field experiments have been undertaken. In mosquito cages in the laboratory, conditions can be controlled and the effects of temperature quantified accurately, but additional causes of mortality experienced in the field, such as predation or disease are absent. The most commonly used method for observing *Aedes* survival in the field is mark-release-recapture (MRR) where marked individuals of a known age are released and the number of recaptures observed over subsequent days [23]. While MRR experiments expose mosquitoes to all causes of mortality in the field, experiments with *Ae. aegypti* and *Ae. albopictus* typically have small sample sizes [24] and sensitivities of spatial mosquito sampling limit the level of survival detail that can be elucidated through the majority of MRR experiments [25]. Furthermore, MRR experiments are limited in number and cover only a narrow range of environmental temperatures (see Additional file 1). There is, therefore, considerable uncertainty over variation in *Aedes* survival in the field.

Using laboratory estimates of survival to predict field survival is complicated by the different mortality risks in the two settings, unknown importance of senescence in the field, and the transition from constant laboratory to fluctuating natural temperature regimes. It has long been assumed that the high rate of mortality in the field, due to external causes, ensures few mosquitoes live long enough to experience age-dependent mortality [26]. Observations from laboratory and field experiments on *Ae. aegypti*, however, have begun to challenge this assumption [23,27]. As a result, the conditions under which age-dependence or age-independence dominates mortality remain unresolved, yet are potentially of considerable epidemiological importance because older mosquitoes are more likely to have survived beyond the virus’s extrinsic incubation period. Furthermore, controlled laboratory conditions are not expected to have the same effect as what is observed in the field. This is particularly apparent with observations at constant temperature in the laboratory in contrast to the fluctuating temperature experienced in the wild. At lower mean temperatures, fluctuation may increase survival because at least part of the day could permit significant increases in survival. The converse is true for optimal mean temperatures, where any positive or negative departure from the optimum could lead to decreased survival [28,29]. Both of these issues have widened the gap between field and laboratory observed survival in terms of the average and distribution of survival times.

Observations from temperature-controlled laboratory experiments have produced a range of candidate parametric functions that are suitable for modelling age-dependent mortality, such as the Gompertz and Logistic functions [27,30]. Early experiments were extended by Degallier *et al.* who observed age-dependent mortality in caged mosquitoes exposed to uncontrolled field temperature regimes [31]. Degallier *et al.* also extended the parametric analysis by using non-parametric Cox proportional hazard models, which allowed them to analyse the significance of both age and environmental conditions on adult mortality. The importance of seasonal changes in temperature was emphasised by Strickman *et al.* who conducted year-round field experiments on caged mosquitoes in Thailand [32]. Yang *et al.* attempted to explicitly model the relationship between temperature and survival, however, their model assumed age-independent mortality and relies on relatively limited data [21]. The paucity of data and the challenges of fitting robust statistical relationships to *Ae. albopictus* adult longevity data are discussed by Waldock *et al.* who highlight the disparity relative to the data available for development of the mosquito’s immature stages at a range of different temperatures [33].

In this study we compiled published observations from a variety of studies and modelled the effect of temperature on survival of adult female *Ae. aegypti* and *Ae. albopictus* under laboratory and field conditions. Our models incorporated the effects of age-dependent mortality and predict survival under fluctuating temperature regimes. They provide a more detailed understanding of *Aedes* adult female mortality and present a statistical solution to investigating age-dependent survival. The resulting models may enhance current DENV transmission models [5,33-36], which could improve predictions that guide vector control, identify areas suitable for DENV transmission and contribute to outbreak early warning systems.

## Methods

### Data collection

Relevant publications were collected by searching the databases of PubMed, Google Scholar and the Armed Forces Pest Management Board Literature Retrieval System using the search terms *Aedes, aegypti, albopictus, survival, mortality, longevity*, *stegomyia* and *albopicta*. Resulting abstracts were examined for their likelihood to contain laboratory-based adult female survival experiments (closed cage with controlled or semi-controlled environmental and feeding regimes). A more detailed inspection of the manuscript allowed us to apply further inclusion criteria for the final database of laboratory survival experiments. Included experiments monitored mosquito survival of at least five individuals at least every two days and gave all mosquitoes access to nutrition (sugar or any form of blood feeding). In the field it appears that female *Ae. aegypti* feed almost exclusively on human blood [37,38], however, laboratory experiments use a range of diets, such as blood plus sugar, sugar only or blood only, artificial blood meals (blood drawn from a variety of different vertebrates presented to mosquitoes in different ways), and direct feeding on a variety of different vertebrates that can confer significant differences in survival [38-43]. As a result this information was collected and incorporated as a random effect in our analysis and all predictions were made for mosquitoes fed human blood. Due to factors other than mosquito diet, laboratory survival observations still showed a significant amount of variation (see Additional file 1), as a result we decided to include a random effect at the experiment level to control for the wide variety of experimental set ups. All experiments analysed used mosquitoes colonised in the laboratory for less than 2 years. Experiments where mosquitoes were chemically or genetically treated were excluded. Finally, *Ae. aegypti* used in the survival experiments were geographically distributed across five continents incorporating both tropical and temperate latitudes. There was a slight bias towards North American samples in the *Ae. albopictus* survival data, however, strains from tropical areas, such as Réunion and Malaysia, were also included.

Literature searches for MRR field experiments were completed independently under a parallel project to investigate mosquito movement (Guerra *et al.* unpublished observations). Briefly, existing literature was searched using a similar key word strategy, adult female mosquito MRR and ancillary geographic data were extracted from suitable articles. Using these references and ancillary data, daily recapture numbers were extracted from relevant articles. Of the *Ae. aegypti* MRR studies, 14% released field caught mosquitoes, 36% released laboratory reared mosquitoes from a recently caught strain and 50% released a colonised mosquito strain. For *Ae. albopictus*, the corresponding breakdown was 4%, 20% and 76%.

Where available, the initial number of mosquitoes (for laboratory experiments) and number of deaths/recaptures on each subsequent day of observation (for MRR experiments) were extracted. Where only graphical summaries were available, these observations were recreated using GetData Graph Digitizer [44]. For any article where observations could not be recorded or recreated the authors were contacted and primary data was requested. For laboratory data the mean temperature was recorded and for field data the maximum and minimum temperatures were recorded. Where unavailable, study site temperatures were estimated using the average climate for the particular location and time of year using WorldClim global climate data [45]. The resulting number of articles and data points included are summarised in Table 1. The full reference list and summary boxplots are available in Additional file 1.

**Table 1:** Summary of laboratory and field data

	***Ae. albopictus *** **laboratory data**	***Ae. albopictus *** **MRR data**	***Ae. aegypti *** **laboratory data**	***Ae. aegypti *** **MRR data**
Number of experiments	210	9	141	50
Mean temperature (^o^C) (±SD)	25.9 (22.3-29.5)	20.3 (17.0-23.7)	25.5 (21.9-29.1)	25.5 (22.9-28.3)
Minimum/Maximum temperature (^o^C)	15/35	16.7/26	10/35	20.7/30.1
Median number of mosquitoes observed/released (IQR)	29 (15–40)	552 (249–1007)	70 (25–382)	602 (493–798)
Dietary regime (%)	Blood: 7.1	-	Blood: 3.0	-
	Sugar: 1.0		Sugar: 38.2	
	Blood + Sugar: 91.9		Blood + Sugar: 58.8	
Age at release (Days) (±SD)	-	3.2 (0.6-5.8)	-	3.6 (1.3-5.9)

SD = Standard deviation, IQR = Interquartile range, MRR = Mark-release-recapture.

### Comparability of data and fitting parametric models to laboratory survival data

A range of parametric equations have been used to model the survival function of laboratory and field adult mosquitoes. For field data, the exponential function, adjusted for recapture number, was the most frequently used model [23,32,46-52]. For laboratory data, a range of functional forms were applied with the aim of detecting age-dependent mortality under conditions where overall mortality is lower. These have included the Gompertz function which can allow a non-symmetric mortality rate to increase or decrease exponentially with age [31,53,54], the Weibull function where the mortality rate is a power function of age [31,48,53,54] and the log-logistic function where the mortality rate is not restricted to a monotonic function [31,54,55]. Here we aimed to compare the fit of these different parametric models to laboratory survival data at each temperature regime across all of the studies to see if any one function offers a significantly improved fit and whether this changes with temperature. Using the laboratory data we re-examined the fit of the log-logistic, Gompertz, exponential and Weibull survival functions. Function fitting was performed in R version 2.14.2 [56] using the “flexsurv” library which fits a variety of parametric models using maximum likelihood. The Akaike Information Criterion (AIC) was calculated for each model and used to calculate the relative likelihood (RL) [57] of each model to the model with the lowest AIC:

$$RL_{j}=e^{\frac{\left(\mathrm{AI}C_{\min}-\mathrm{AI}C_{j}\right)}{2}}$$

The relative likelihood values for each model were averaged for experiments of equivalent temperature to give a balanced estimate of the each model’s suitability at different temperatures.

### Fitting non-parametric models to laboratory survival data

As the distribution of survival times may undergo complex changes across a range of temperatures we also chose to construct a non-parametric model that would not share the same restrictions as the aforementioned parametric models. For this we chose regression spline generalised additive models (GAMs) which apply a smoothing variable to the explanatory variables in order to model the response variable [58]. This method has the advantage of being able to model unknown and non-linear effects of covariates and thus elucidate the potentially complex effect of temperature on adult mosquito survival.

To evaluate the improvement of using GAMs over the parametric alternatives, we fitted parametric and GAM models to each laboratory experiment and calculated the difference in AIC between parametric and non-parametric models across all experiments.

A second GAM was then formulated to use the data from all experiments in one model to recreate the relationship between survival, time and temperature. The GAM was formulated as follows:

$$S_{\mathrm{ij}}\sim \mathrm{Binomial}\;\left(N_{\mathrm{ij}},p_{\mathrm{ij}}\right)$$

$$\mathrm{logit}\;\left(p_{\mathrm{ij}}\right)=f\left(D_{i},T_{i}\right)+\epsilon_{j}+\epsilon_{d}$$

$$\epsilon_{j}\sim N\;\left(0,{\theta_{j}}^{2}\right)$$

$$\epsilon_{d}\sim N\;\left(0,{\theta_{d}}^{2}\right)$$

*S*_*ij*_ = number of mosquitoes surviving at observation *i* in experiment *j*

*N*_*ij*_ = number of mosquitoes at start of time step at *i, j*

*P*_*ij*_ = survival probability for a mosquito at *i, j*

*f*() = smooth term

*D*_*i*_ = day of observation *i*

*T*_*i*_ = temperature of observation *i*

*ϵ*_*j*_ = random error term for experiment *j*

*ϵ*_*d*_ = random error term for mosquito diet *d*

*θ*_*j*_^*2*^ = variance across experiments

*θ*_*d*_^*2*^ = variance across mosquito diets

Smoothing parameters were selected by restricted maximum likelihood with a data-driven basis dimension choice of *k*_*D*_ *=* 8 and 5 and *k*_*T*_ = 5 and 5 for *Ae. aegypti* and *Ae. albopictus* respectively [59]. Confidence intervals for the interquartile range of predictions were obtained by bootstrapping with 200 repeats, each the size of the original dataset. This model was fit using *D* ≥ 1 to be consistent with the experimental observations that record mortality. Extrapolated model predictions for 0 ≤ *D* < 1 were scaled proportionally to ensure 100% survival at *D* = 0. All GAMs were implemented using the “mgcv” package in R [60].

For the model to fit biologically appropriate responses, additional data defining the limits of prediction were required. Observations from Christophers [18], suggest 4°C and 42-43°C as suitable minimum and maximum critical temperatures at which survival of *Ae. aegypti* is minimal (<24 hours). Similar observations for *Ae. albopictus* suggest values between -5°C and 40–40.6°C [61,62]. To constrain mortality in the model, all non right censored experimental observations were extended to 120 days at 0% survival and a maximum lifetime of 120 days was imposed at all temperatures, the maximum longevity observed in our dataset. Furthermore, to produce meaningful estimates of longevity, survival of less than 0.1% of the initial mosquito population was considered sufficient to indicate complete mortality.

### Reconciling laboratory survival predictions with survival measurements in the field

**Figure 1:**
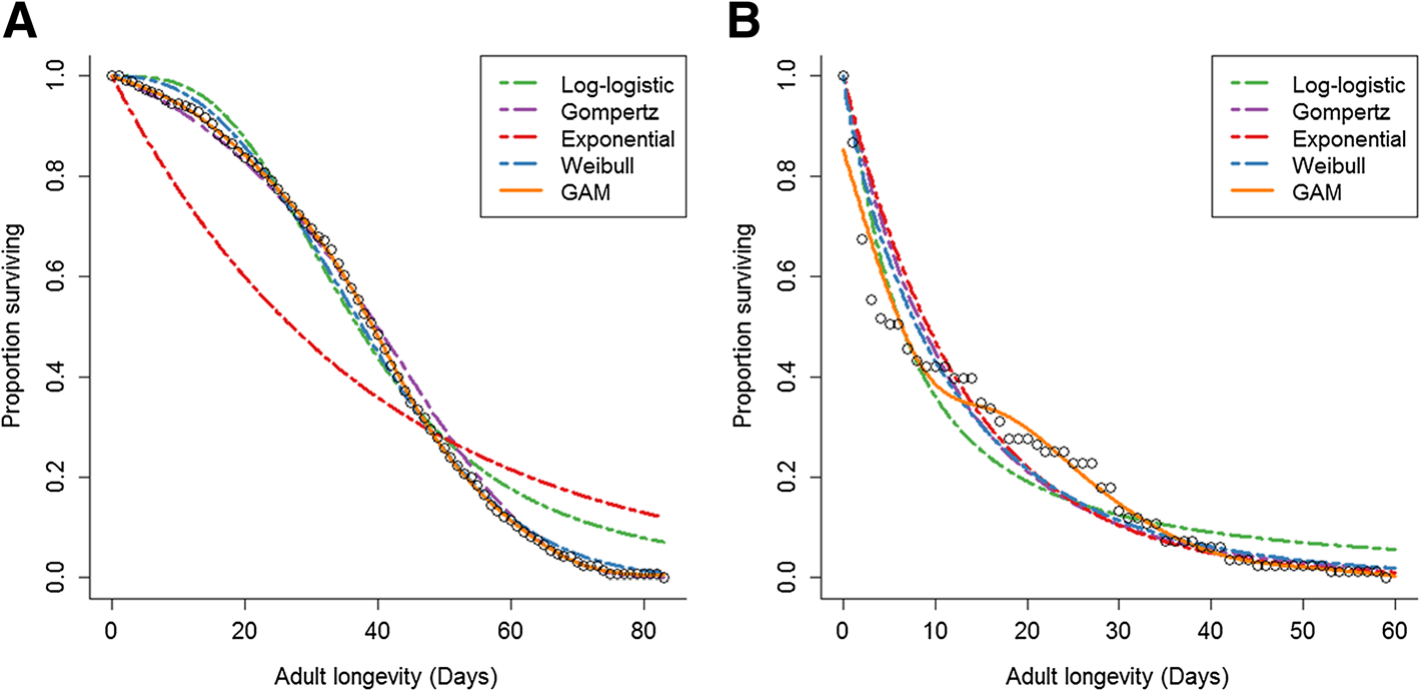
**Schematic overview of the methods.** Green parallelograms indicate input data, orange rectangles show processing or modelling steps, blue diamonds show decision steps and red boxes show output analysis and models (dotted shows intermediate, unbroken line shows final outputs). MRR = Mark-release-recapture.

Because MRR data was too limited to fit models over a range of temperatures as we did for the laboratory mosquitoes, we used the laboratory-based models above to estimate temperature and age-matched expected mortality rates for mosquitoes in the field and compared those estimates to observed field mortality estimates from MRR data. To simulate the realistic effects of temperature in the field, in contrast to the constant temperatures of the laboratory, we recreated 59 separate daily fluctuating temperature regimes using the maximum and minimum temperatures of each MRR experiment and assumed sinusoidal progression in the day with a decreasing exponential curve at night [28,63]. These matching fluctuating temperatures were then applied to the laboratory survival model to estimate hourly survival for each of the MRR experiments. For each MRR experiment, we directly estimated the hourly mortality rate using the Buonaccorsi nonlinear regression method applied using the “stats” package in R [60]. This assumes that the mortality rate in the field is constant for each experiment and, therefore, our estimation of external mortality, due to causes such as predation or disease, also has this assumption. We then matched the laboratory-based predicted mortality with the directly estimated mortality and calculated the average difference, which we attribute to mortality due to external factors. We added this estimated hourly external mortality to the temperature-dependent laboratory model to produce the field survival model, which estimates mortality at all temperatures. Confidence intervals for the field survival model were derived by propagating the uncertainty of the laboratory model and combining this with 200 bootstrap samples of the MRR experiments to test the sensitivity of the calculated external mortality value. The overall schematic of the methods appears in Figure 1.

## Results

### Parametric models of laboratory data

**Figure 2:**
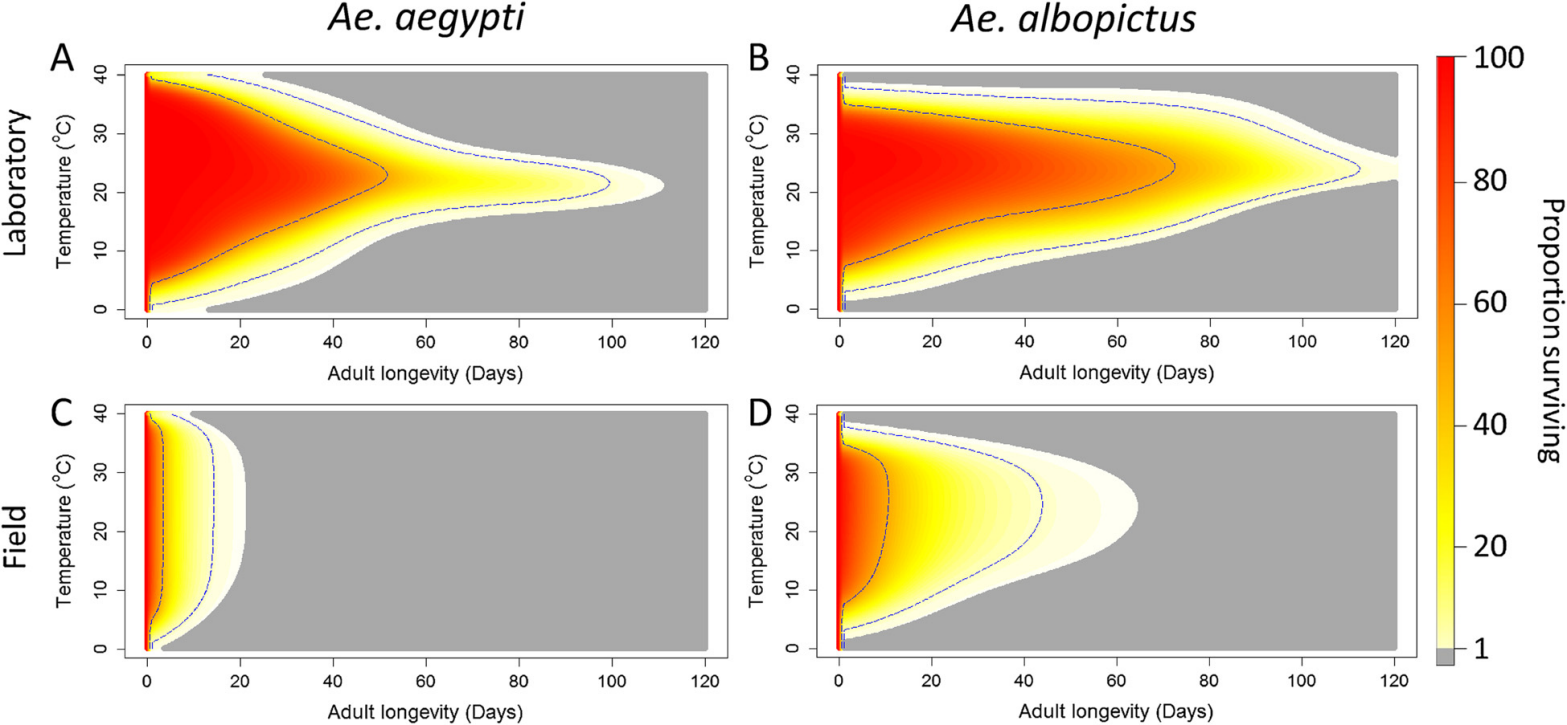
**Relative likelihood of four different parametric models for *Aedes *adult female survival data over a range of constant temperatures.** The models included are i) a two parameter Log-logistic model (shape and scale), ii) a two parameter Gompertz model (shape and rate), iii) a one parameter Exponential model (rate) and iv) a two parameter Weibull model (shape and scale).

Of the four parametric models tested, no one model was consistently most suitable across the range of temperatures tested (Figure 2). The log-logistic model was most suitable for *Ae. aegypti* with a relative likelihood of 0.441, whereas the exponential model was most suitable for *Ae. albopictus* with a relative likelihood of 0.397. The Weibull model was the least suitable for both species with relative likelihoods of 0.07 and 0.05 for *Ae. aegypti* and *Ae. albopictus*, respectively. While some models may be more suitable at specific temperatures, such as the exponential at 15°C for *Ae. aegypti*, they are not consistently more suitable at other temperatures, nor is there a clear trend suggesting some models are more suitable at higher or lower temperatures (Figure 2).

### Non-parametric models of laboratory data

**Figure 3:**
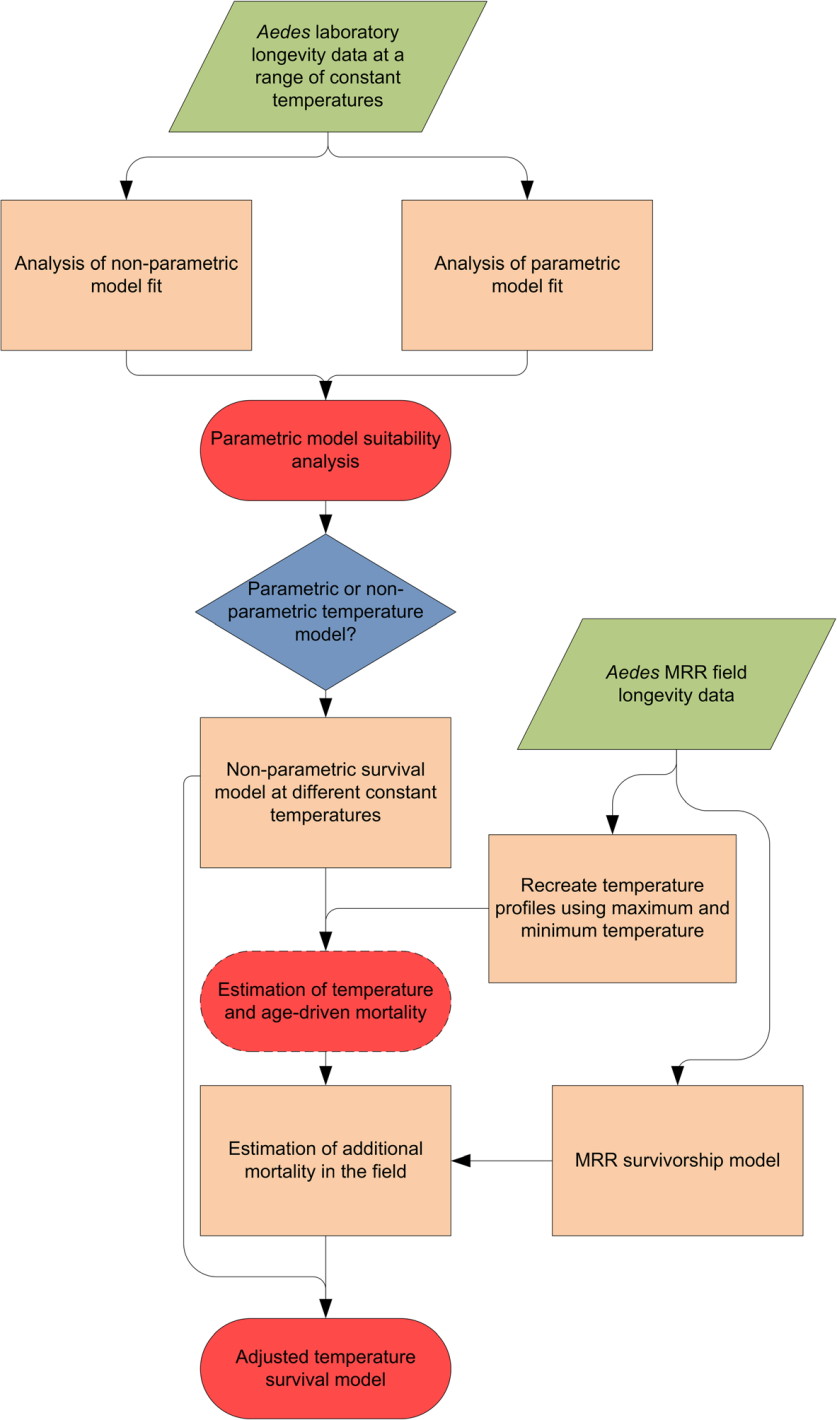
**Examples of parametric and non-parametric model fit.** Open circles show *Ae. aegypti* survival data under controlled laboratory conditions from Joy *et al.*[64]**(A)** and Yang *et al.*[21]**(B)**, two experiments that show contrasting survival curve shape. Parametric models are shown as dashed lines and the non-parametric GAM is shown as a solid orange line.

When non-parametric GAMs were used to fit the same data, both the overall model likelihood and the number of experiments for which it was optimal increased (Table 2). For both *Ae. aegypti* and *Ae. albopictus*, respectively, median difference in AIC between parametric and non-parametric models was 307.6 and 4.38. While still significant, this improvement was less pronounced in *Ae. albopictus* datasets due to a lower average number of mosquitoes under observation (Table 1), which limited the ability of some experiments to detect finer scale changes in survival. The number of experiments for which GAMs were the most suitable, however, (49.5%, Table 2) showed its improved fit over the entire dataset. The fits of both parametric and non-parametric models to two of the *Ae. aegypti* experiments are shown in Figure 3 as an example of the fit of each of the five models. Due to its more accurate quantification of mortality rate and its improved fit across a range of temperatures we chose a non-parametric GAM to construct our laboratory temperature survival model.

**Table 2:** Evaluation of parametric and non-parametric model fit to laboratory data

**Model**	**Median increase in AIC relative to GAM**	**Optimal model choice (%)**
	**( *Ae. aegypti */ *Ae. albopictus *)**	**( *Ae. aegypti */ *Ae. albopictus *)**
Log-Logistic	320.40/22.620	4.4/1.5
Gompertz	304.83/0.005	2.2/21.9
Exponential	302.89/2.969	1.5/13.8
Weibull	302.90/0.023	0.7/13.3
GAM	-	91.1/49.5

AIC = Akaike information criterion, GAM = Generalised additive model.

**Figure 4:**
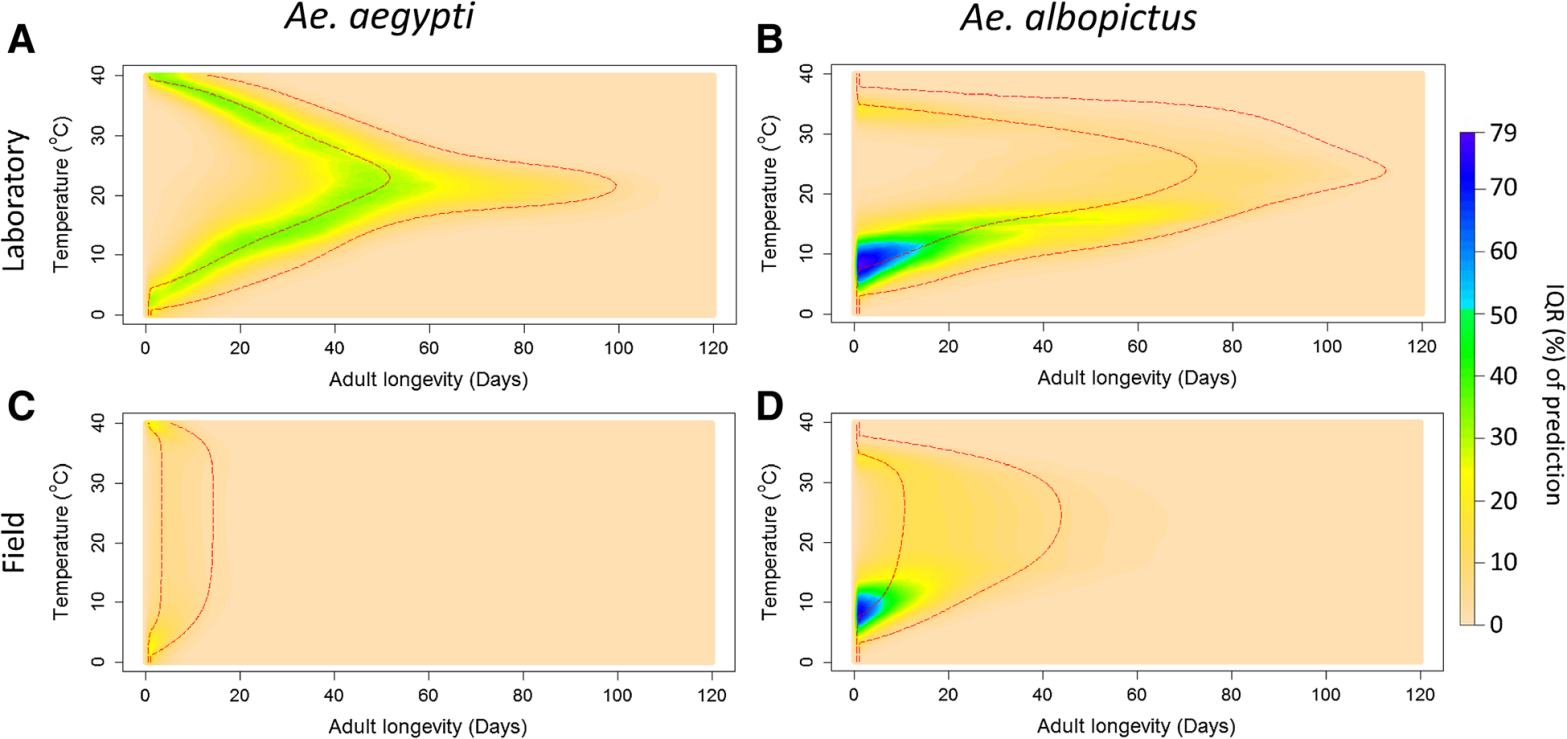
**The distribution of adult female *Aedes aegypti *and *Aedes albopictus *survival across a range of temperatures under laboratory conditions (A and B) and field conditions (C and D).** Colours from red to yellow show survival from 100% - 1% of the population remaining. Grey indicates <1% of the population remaining. Dotted blue lines show the limits for 50% and 95% of the original population remaining.

The non-parametric GAM model allowed us to quantify the effects of age and temperature on mortality whilst still taking into account the random effects at the experiment and mosquito diet level. Figures 4A and 4B show the model fit for each species using the laboratory data. Overall, *Ae. albopictus* had a greater longevity than *Ae. aegypti*, however, *Ae. aegypti* tolerated a wider range of temperatures (Figures 4A and 4B). The survival patterns also differ at the limits of survival, *Ae. aegypti* had optimal survival over only a narrow window around 21°C, while *Ae. albopictus* had optimal survival over a much wider range with only minor differences observed between 20-30°C. The mean and 95% survival limits for both species show that mortality was widely distributed across different ages with the majority of mosquitoes dying well before reaching maximum longevity. For *Ae. aegypti* the predicted median longevity was only 38 days even at optimal temperatures despite some individuals living over 100 days. This same pattern was observable in *Ae. albopictus* data, but to a lesser extent: median longevity at optimal temperatures was over 60 days despite some individuals living up to 120 days. Random effects for mosquito diet and study level were highly significant for both *Ae. aegypti* and *Ae. albopictus* models (Chi square, p < 0.001).

**Figure 5:**
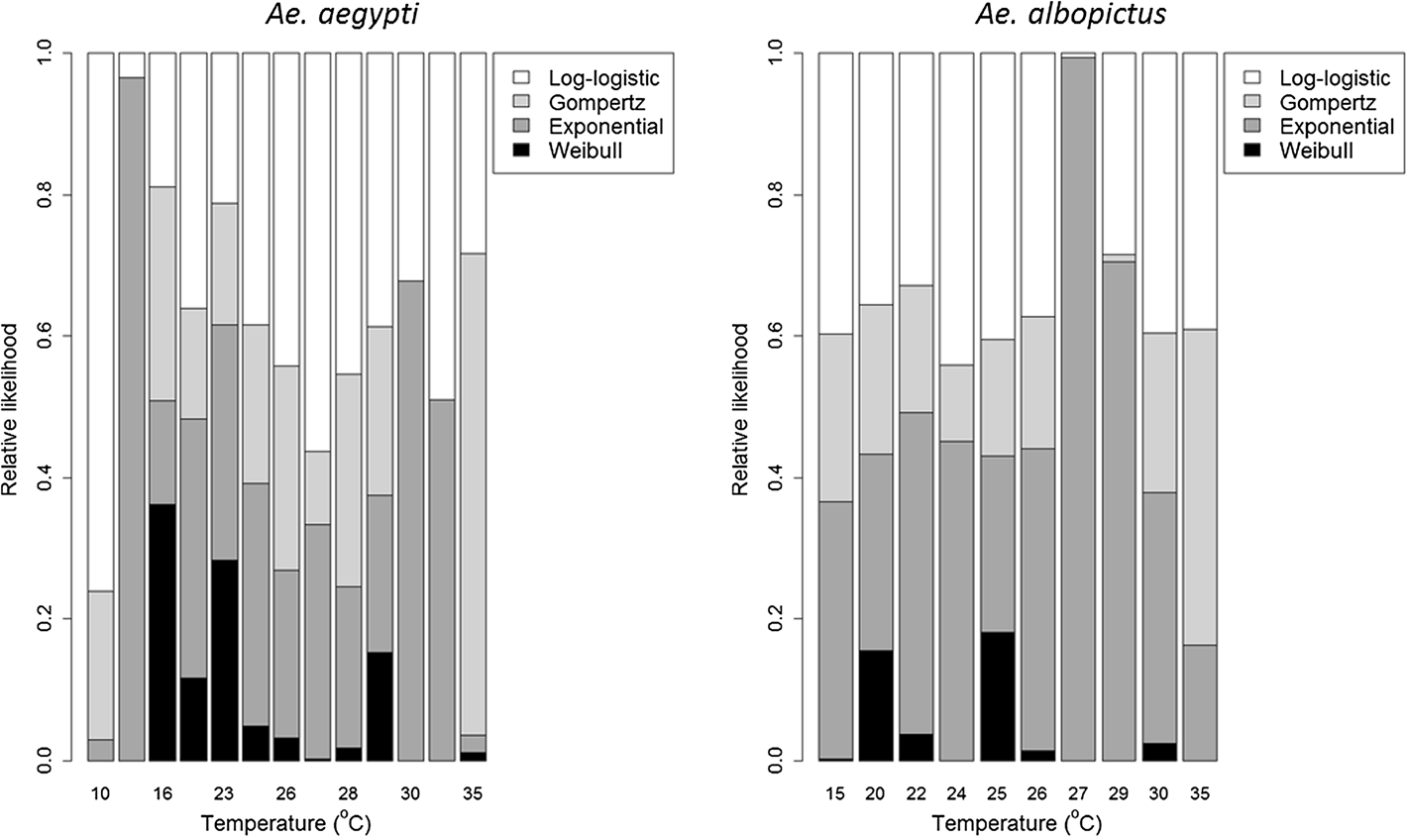
**The distribution of uncertainty of the laboratory model prediction.** Colours from blue to beige show the interquartile range (IQR) in predictions from 200 bootstrap runs of the laboratory model **(A and B)**. This uncertainty is then combined with the field data uncertainty quantified by 200 bootstrap runs of MRR data to give the IQR predictions for the field survival model **(C and D)**. Red dotted lines of the 50% and 95% of the population remaining are added for reference.

The greatest uncertainty around these predictions occurs around the 50% survival time (Figure 5A and 5B). For the majority of survival curves this is the point at which survival has the greatest rate of change; i.e. highest mortality rate, and it is, therefore, not surprising that uncertainty is higher around these points. Peaks in uncertainty can also be identified at low temperatures for *Ae. albopictus* (Figure 5B). Overall the uncertainty was higher for *Ae. albopictus* reflecting the limited data, particularly between 0 and 15°C.

### Reconciliation with field data

To estimate longevity in the field, the daily mortality in the laboratory model was compared to field observations of mortality from MRR experiments where other, external factors may contribute to mortality. For *Ae. aegypti*, the laboratory model-predicted daily mortality rate varied between 0.05 and 0.082 (average 0.064) compared to an observed MRR mortality rate of between 0.033 and 0.595 (average 0.288). The average difference between these predictions for each experiment, and thus the calculated additional daily mortality, was 0.179 (standard deviation 0.012 – 0.346). For *Ae. albopictus* the external daily mortality was lower. Laboratory model predictions of daily mortality rate varied from 0.0024 to 0.289 (average 0.040) compared to observed MRR mortality rate of between 0.031 and 0.231 (average 0.121) giving an average difference of 0.0621 (standard deviation −0.030 – 0.154). Due to the techniques used to measure external mortality, we had to assume it acted independently of mosquito age or temperature and so predicted survival in the field was calculated as the sum of laboratory model mortality and external mortality and is shown in Figure 4C and 4D.

Clear differences in survival can be observed between laboratory models (Figure 4A and 4B) and field models (Figure 4C and 4D). Survival is decreased for both species, but less so for *Ae. albopictus* due to the lower estimated external mortality. The effect of incorporating external mortality also reduces the relative influence of temperature and as a result less variation in survival is observed at mid-range temperatures (approximately 15-35°C). The most significant difference between laboratory and field models is the change in median longevity, which is reduced considerably in both species.

When field data uncertainty was incorporated with the existing laboratory model uncertainty, overall uncertainty was reduced due to the higher value of external mortality and the lower uncertainty in estimating this parameter (Figure 5). Uncertainty was higher at extreme temperatures, particularly for *Ae. albopictus* at lower temperatures, however this uncertainty was confined to a limited range of temperatures and days, ensuring that overall prediction had low uncertainty. The predicted hourly survivorship from the models for *Ae. aegypti* and *Ae. albopictus* field survival and their associated uncertainty profiles are available for free download on the online data repository figshare (http://www.figshare.com) [65,66].

## Discussion

In this study we used data from 410 *Ae. aegypti* and *Ae. albopictus* adult female survival experiments to construct a model of temperature and age-dependent survival. The best-fitting model was an age and temperature dependent GAM, which we combined with field-based mortality to estimate the distribution of longevity of mosquitoes in the field. These models should be interpreted in light of the limitations to the data used and the modelling methods. Of the data compiled, there were relatively few experiments conducted at more extreme temperatures, resulting in higher uncertainty in these temperature ranges. Considering the implications these extreme temperatures have for the geographic range of the mosquito, more data is necessary for better defining that range. Mosquito survival may also vary geographically due to variation in the genetic background of local mosquito populations. Our analysis incorporates experimental data from a wide variety of locations and conditions but, given the paucity of MRR data at climatic extremes, it is possible that estimates of field-based mortality may not be representative, particularly in locations where mosquitoes may be more tolerant of extreme climates. However, observations suggest that adult survival is one of the few bionomics of *Ae. aegypti* that remains consistent among geographically disparate populations [67,68]. North American samples of *Ae. albopictus* were over-represented in our laboratory data (91%) which may overestimate thermal tolerance at the lower temperature limits due to evidence of *Ae. albopictus* adaptation in temperate climates [53,69]. Perhaps the most important data limitation is that there are very few field mortality experiments upon which to estimate mortality outside of the laboratory, where arbovirus transmission actually occurs. More data would allow better estimation of field mortality and allow relaxation of the assumption that external contributors to mortality are not age-related. An extension should include alternatives to MRR approaches that might be more sensitive for estimating survival of older potentially infectious mosquitoes, for example, using transcript levels of an age-associated gene [70] or measuring mosquito infection rates [71].

It is also worth considering the scope of the model we developed. While it may suggest that adult survival is possible even at low temperatures, factors limiting the development of the immature stages may preclude the establishment of an adult population. For adult *Aedes*, there may also be additional temperature-based limits to survival. Below 14-15°C, *Ae. aegypti* experiences reduced mobility and struggles to imbibe blood [18,21,72]. As observations (at tropical temperatures) suggest *Ae. aegypti* cannot survive longer than 2-3 days without a blood meal [73], extended periods of time below 14-15°C are likely to result in complete mortality. We extended our predictions below these temperatures because limited time spent at these colder temperatures, such as a few hours in the early morning, may not confer a lasting negative effect on survival, however, these effective mortality limits must be considered when integrating this into a wider *Aedes*/DENV transmission model.

Here we have shown that GAMs captured more of the variation in survival between different experiments than conventional, parametric models of mosquito mortality, including the Gompertz and logistic models which previously provided the best fit of the options examined [27,30,31]. In our analysis we did not consider frailty-based mortality models, however non-parametric approaches, such as GAM, offer more flexibility than parametric models, suitable predictive power and straight-forward implementation in a variety of statistical software packages. We have also emphasised the importance of including random effects in analyses of this type as it has allowed us to comment on the effect of specific experimental treatments, such as feeding regime, and thus guide laboratory and field experimental design. Furthermore adding a random effect at the study level allowed us to comment on the generalisability of any one experiment to a wider context.

The relative fit of the GAM indicated the presence of age-dependent survival. This age-related effect may be related to senescence or other factors, but appears to be less important in the wild. On average we found that mortality due to external causes was significantly greater (4.5 times for *Ae. aegypti* and 3.0 times for *Ae. albopictus*) than mortality due to temperature and age at mid-ranging temperatures (20-30°C) indicating that few mosquitoes will be alive at older ages by the time senescence measurably impacts mortality. Results from our analysis indicate that across different temperature regimes the assumption of age-independent mortality for *Ae. aegypti* and *Ae. albopictus* in the field is likely to be appropriate. It may be possible that senescence acts at different ages in the laboratory than it does in the field, however, quantifying this would require unfeasibly large mosquito cohorts, or innovative new techniques to measure mosquito survival in the field. As a result, the statistical model presented here provides a novel approach for quantifying the effects of age-dependent survival among wild mosquitoes.

The greater tolerance of lower temperatures observed for *Ae. aegypti* compared to *Ae. albopictus* appears at odds with their observed geographic distribution. This finding can be explained, however, if we consider the adaptations of the egg stages of each mosquito. *Aedes albopictus* eggs are able to undergo diapause allowing the species to persist during cold winter temperatures that are unfavourable to adult survival [74,75]. *Aedes aegypti* shows only limited adaptation to egg stage survival in unfavourable periods [76,77] and there may, therefore, be greater selection pressure for thermal tolerance at the adult stage to resist diurnal and inter-seasonal variations in temperature.

The seasonal variation and geographic limits of *Aedes* transmitted viruses are intrinsically linked to the seasonality and geographic limits of mosquito populations required for their transmission. It is, therefore, important that the survival of adult *Aedes* is well estimated using appropriate data and considering the variety of conditions they may encounter in nature. Quantifying how changes in survival translate to dengue transmission potential is also important and requires integration with models of the DENV extrinsic incubation period, which is also sensitive to temperature [78]. Characterising the interaction between mortality and extrinsic incubation could be used further to explore entomological components of seasonal forcing among *Aedes*-borne viruses, which may help inform strategies for disease prevention and control.

Given that dengue is a disease that affects over half the world’s population [12] with an estimated 390 million new infections per year [10], improvements in existing approaches and new strategies to control the disease are needed. Depending on the situation, both the type of vector control used and how it is implemented must be optimised to achieve the biggest reduction in dengue burden. Modelling and mapping are two important tools for addressing both of these issues, identifying risks and opportunities for control [34,79,80]. Considerable efforts have already been undertaken to re-examine many of the fundamental assumptions of early *Aedes* and dengue transmission models [6,78,81,82]. The next generation of models is likely to incorporate significant advances, including new frameworks for modelling survival of adult mosquitoes. The adult *Aedes* survival models developed here can be integrated into these new modelling approaches with the aim of refining the global distribution of *Aedes* vectors, guiding seasonal vector control efforts, modelling dengue transmission and developing early warning systems to prevent dengue epidemics [83].

## Conclusions

We created an explicit model of *Ae. aegypti* and *Ae. albopictus* adult survival across a range of temperatures under both laboratory and field conditions. Commonly used parametric models did not capture the variation in survival to the same extent as our approach across a variety of temperatures and experiments. The use of non-parametric GAMs allowed the effects of senescence and temperature to be captured explicitly, revealing the importance of temperature and age-dependent mortality under laboratory conditions and fluctuating temperatures and external mortality in the field. The models developed here can be used for investigating seasonal variation in dengue transmission and will strengthen components to dengue transmission models that investigate various options for control.

## Abbreviations

DENV: Dengue virus; MRR: Mark-release-recapture; AIC: Akaike’s information criterion; RL: Relative likelihood; GAM: Generalised additive model.

## Competing interests

Disclaimer: The findings and conclusions are those of the authors and do not necessarily represent the official position of the Centers for Disease Control and Prevention.

## Authors’ contributions

OJB designed the experiment, wrote the manuscript and collected and analysed the data. MAJ, DLS, TWS, PWG and SIH also helped conceive and design the experiments. MAJ, SB, NG, DMP, DLS and TWS helped with data analysis and interpretation. CAG, HD, MGG, PL, RM and LMS collected data. All authors were involved in drafting and revising the manuscript and all authors approved the final version.

## Supplementary Material

Additional file 1 Data bibliography and summary statistics. Click here for file
