# Supplementary material for: Modelling adult Aedes aegypti and Aedes albopictus survival at different temperatures in laboratory and field settings
Source: Parasit Vectors. 2013 Dec 12;6:351. doi: 10.1186/1756-3305-6-351 (PMC3867219; doi:10.1186/1756-3305-6-351)
Supplement: Additional file 1 — Data bibliography and summary statistics. [file 1756-3305-6-351-S1.pdf]

# Additional file 1, data bibliography and summary statistics

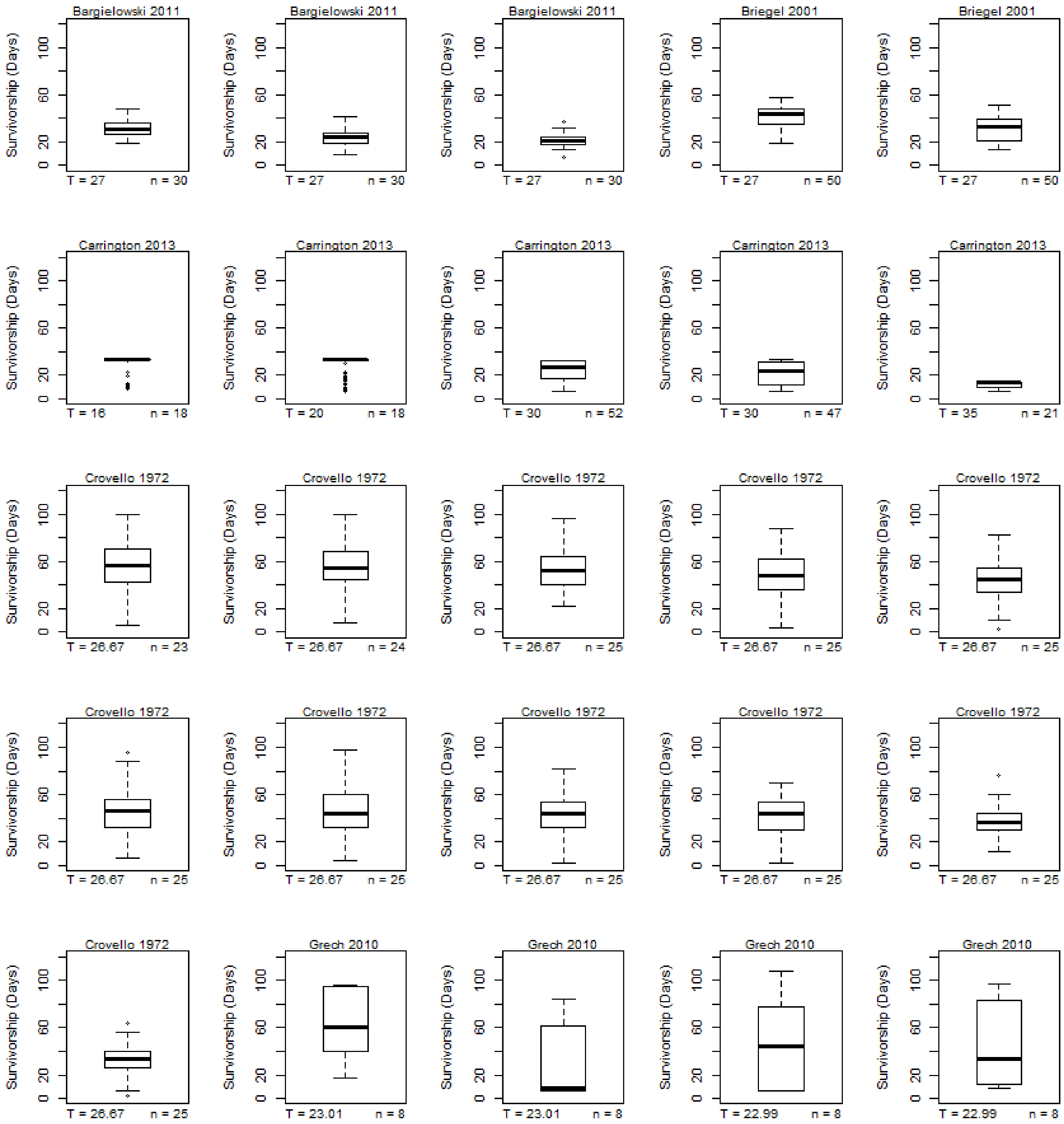

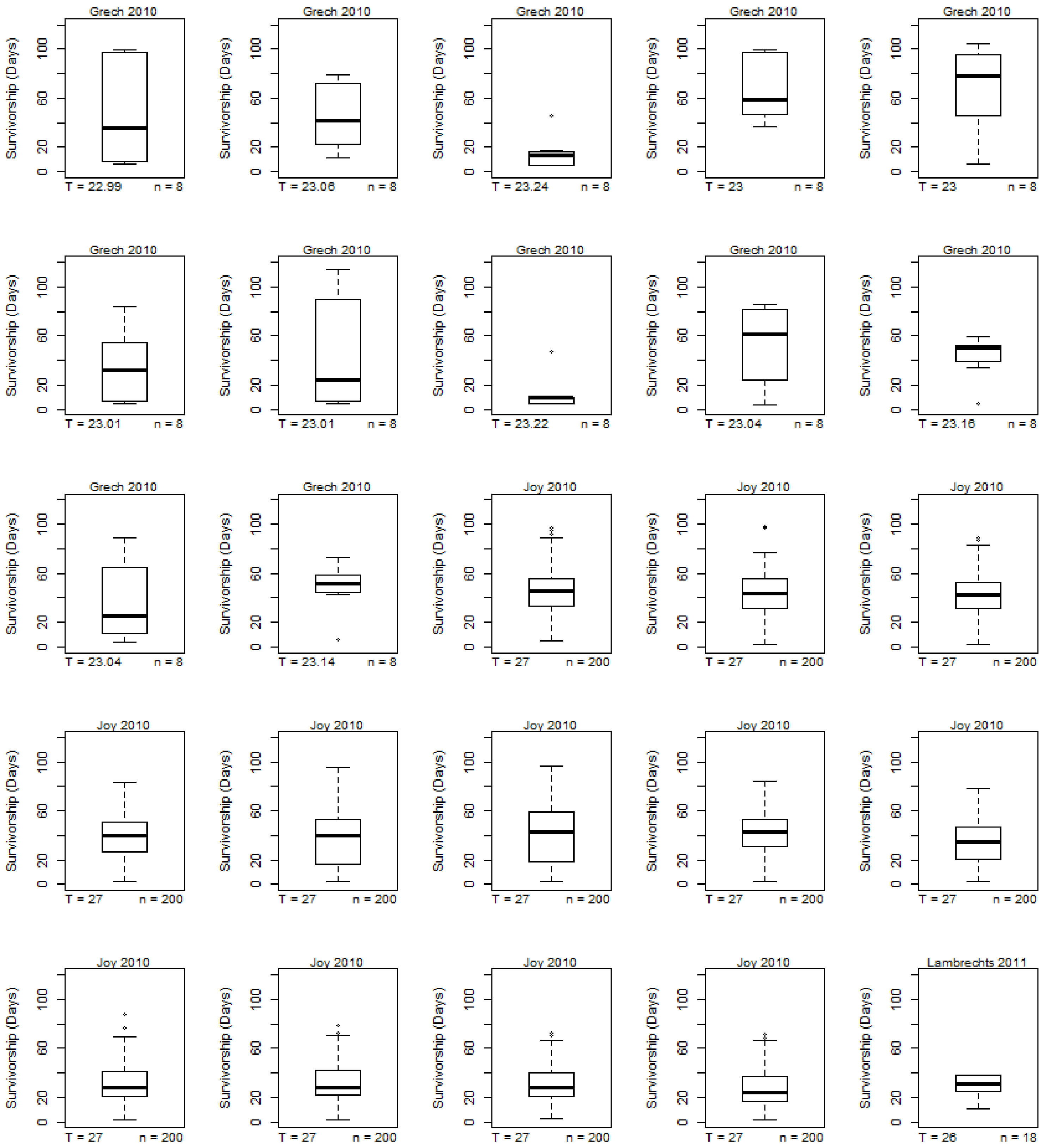

Grech 2010
Survivorship (Days)
0
20
60
100
T = 22.99
n = 8
Grech 2010
T = 23.06
n = 8
Grech 2010
T = 23.24
n = 8
Grech 2010
T = 23
n = 8
Grech 2010
T = 23
n = 8
Grech 2010
T = 23.01
n = 8
Grech 2010
T = 23.01
n = 8
Grech 2010
T = 23.22
n = 8
Grech 2010
T = 23.04
n = 8
Grech 2010
T = 23.16
n = 8
Grech 2010
T = 23.04
n = 8
Grech 2010
T = 23.14
n = 8
Joy 2010
T = 27
n = 200
Joy 2010
T = 27
n = 200
Joy 2010
T = 27
n = 200
Joy 2010
T = 27
n = 200
Joy 2010
T = 27
n = 200
Joy 2010
T = 27
n = 200
Joy 2010
T = 27
n = 200
Joy 2010
T = 27
n = 200
Joy 2010
T = 27
n = 200
Joy 2010
T = 27
n = 200
Joy 2010
T = 27
n = 200
Joy 2010
T = 27
n = 200
Lambrechts 2011
T = 26
n = 18

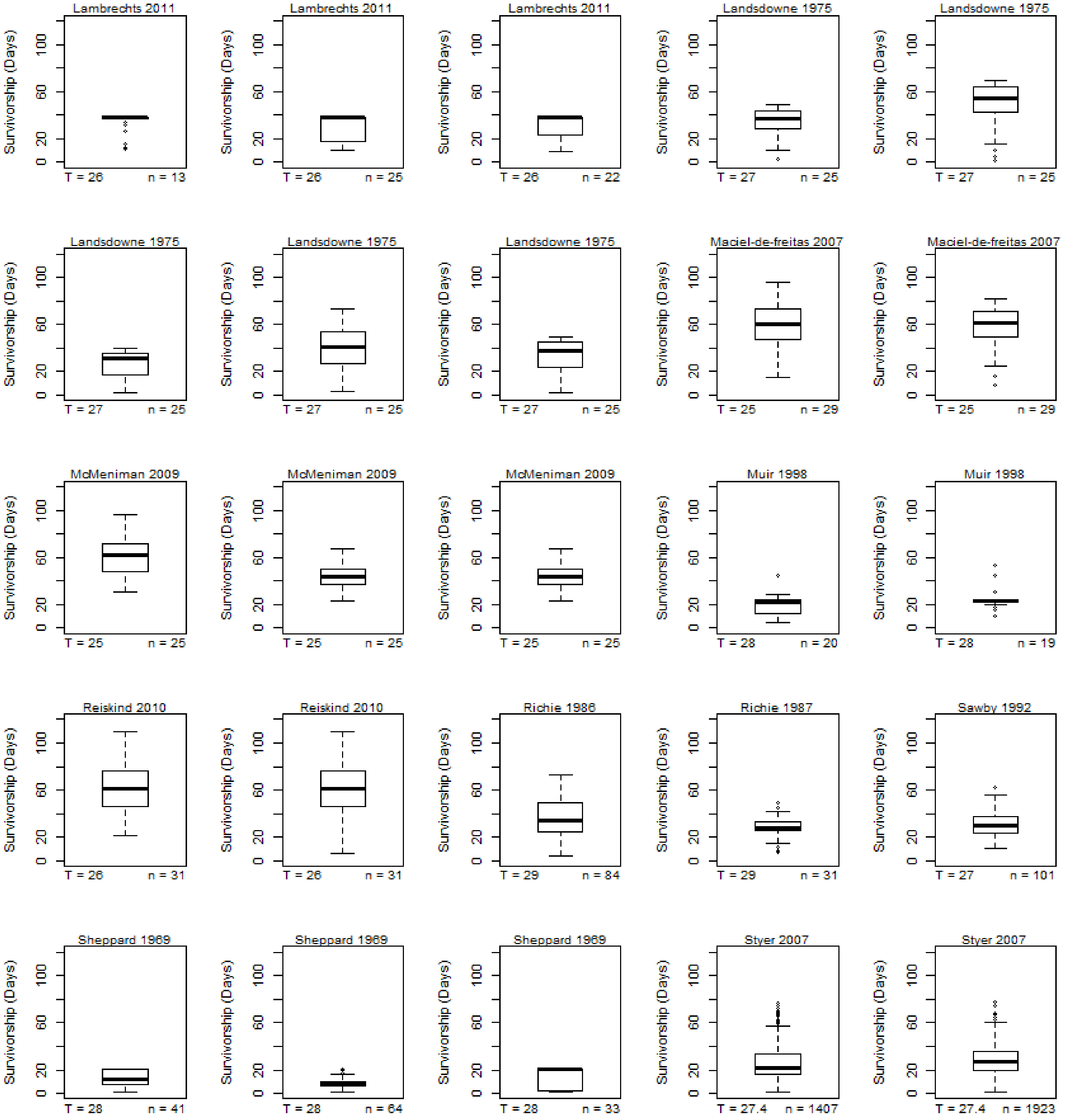
Lambrechts 2011
T = 26 n = 13
Lambrechts 2011
T = 26 n = 25
Lambrechts 2011
T = 26 n = 22
Landsdowne 1975
T = 27 n = 25
Landsdowne 1975
T = 27 n = 25
Landsdowne 1975
T = 27 n = 25
Landsdowne 1975
T = 27 n = 25
Landsdowne 1975
T = 27 n = 25
Maciel-de-freitas 2007
T = 25 n = 29
Maciel-de-freitas 2007
T = 25 n = 29
McMeniman 2009
T = 25 n = 25
McMeniman 2009
T = 25 n = 25
McMeniman 2009
T = 25 n = 25
Muir 1998
T = 28 n = 20
Muir 1998
T = 28 n = 19
Reiskind 2010
T = 26 n = 31
Reiskind 2010
T = 26 n = 31
Richie 1986
T = 29 n = 84
Richie 1987
T = 29 n = 31
Sawby 1992
T = 27 n = 101
Sheppard 1969
T = 28 n = 41
Sheppard 1969
T = 28 n = 64
Sheppard 1969
T = 28 n = 33
Styer 2007
T = 27.4 n = 1407
Styer 2007
T = 27.4 n = 1923
Survivorship (Days)
0
20
60
100

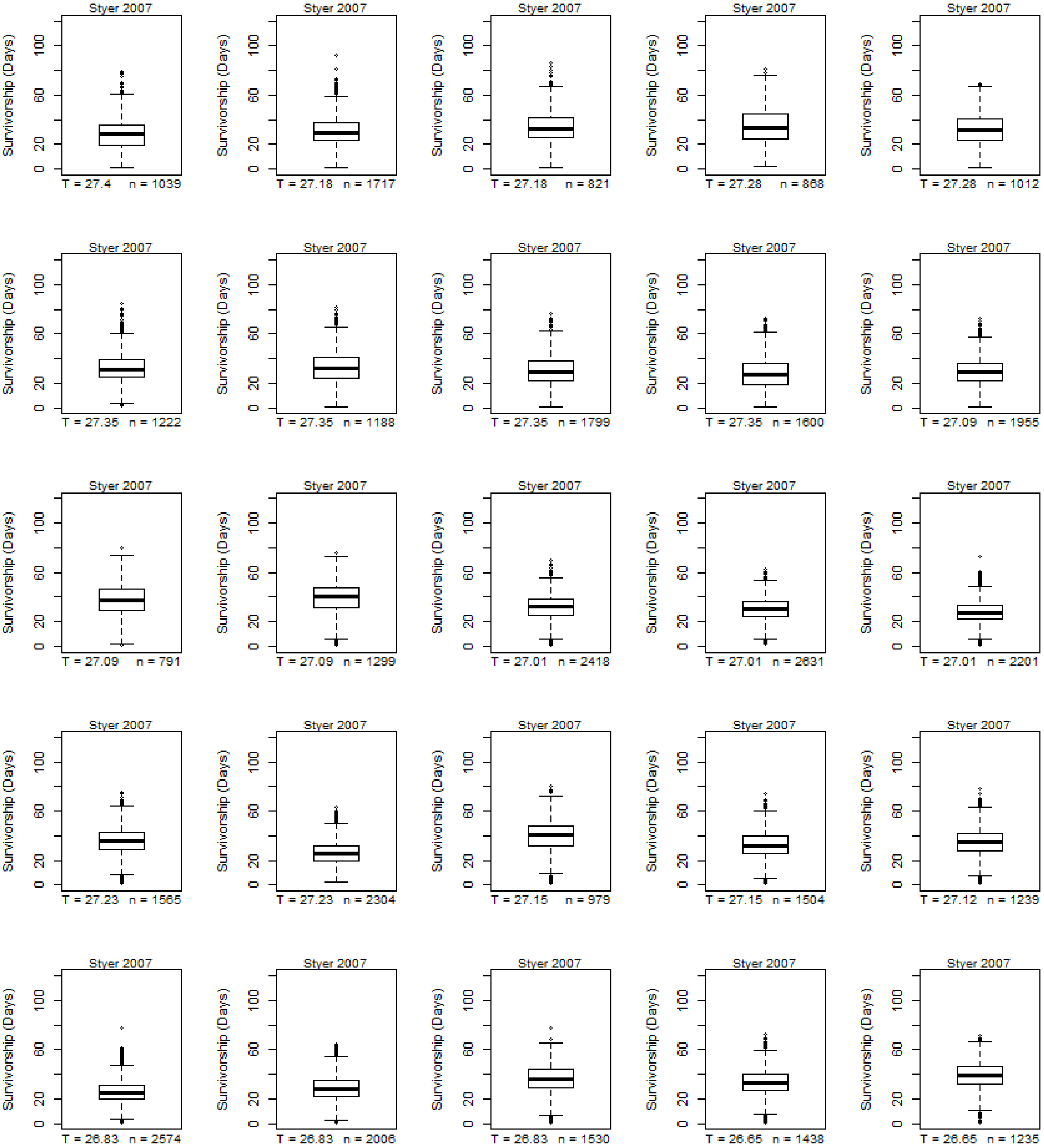
Styer 2007
Survivorship (Days)
0 20 60 100
T = 27.4 n = 1039
Styer 2007
T = 27.18 n = 1717
Styer 2007
T = 27.18 n = 821
Styer 2007
T = 27.28 n = 868
Styer 2007
T = 27.28 n = 1012
Styer 2007
T = 27.35 n = 1222
Styer 2007
T = 27.35 n = 1188
Styer 2007
T = 27.35 n = 1799
Styer 2007
T = 27.35 n = 1600
Styer 2007
T = 27.09 n = 1955
Styer 2007
T = 27.09 n = 791
Styer 2007
T = 27.09 n = 1299
Styer 2007
T = 27.01 n = 2418
Styer 2007
T = 27.01 n = 2631
Styer 2007
T = 27.01 n = 2201
Styer 2007
T = 27.23 n = 1565
Styer 2007
T = 27.23 n = 2304
Styer 2007
T = 27.15 n = 979
Styer 2007
T = 27.15 n = 1504
Styer 2007
T = 27.12 n = 1239
Styer 2007
T = 26.83 n = 2574
Styer 2007
T = 26.83 n = 2006
Styer 2007
T = 26.83 n = 1530
Styer 2007
T = 26.65 n = 1438
Styer 2007
T = 26.65 n = 1235

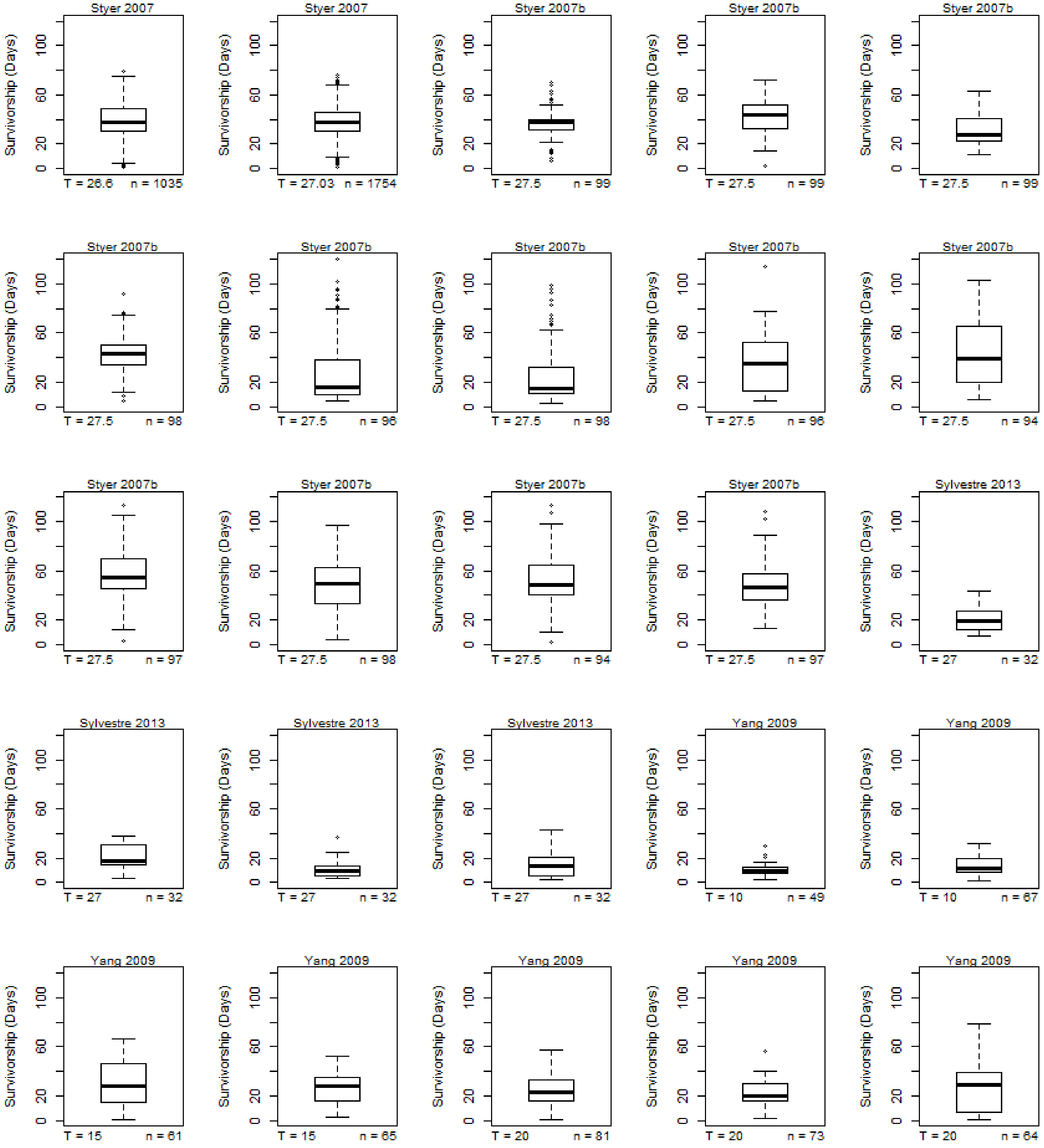
Survivorship (Days)
Styer 2007
T = 26.6 n = 1035
Styer 2007
T = 27.03 n = 1754
Styer 2007b
T = 27.5 n = 99
Styer 2007b
T = 27.5 n = 99
Styer 2007b
T = 27.5 n = 99
Styer 2007b
T = 27.5 n = 98
Styer 2007b
T = 27.5 n = 96
Styer 2007b
T = 27.5 n = 98
Styer 2007b
T = 27.5 n = 96
Styer 2007b
T = 27.5 n = 94
Styer 2007b
T = 27.5 n = 97
Styer 2007b
T = 27.5 n = 98
Styer 2007b
T = 27.5 n = 94
Styer 2007b
T = 27.5 n = 97
Sylvestre 2013
T = 27 n = 32
Sylvestre 2013
T = 27 n = 32
Sylvestre 2013
T = 27 n = 32
Sylvestre 2013
T = 27 n = 32
Yang 2009
T = 10 n = 49
Yang 2009
T = 10 n = 67
Yang 2009
T = 15 n = 61
Yang 2009
T = 15 n = 65
Yang 2009
T = 20 n = 81
Yang 2009
T = 20 n = 73
Yang 2009
T = 20 n = 64

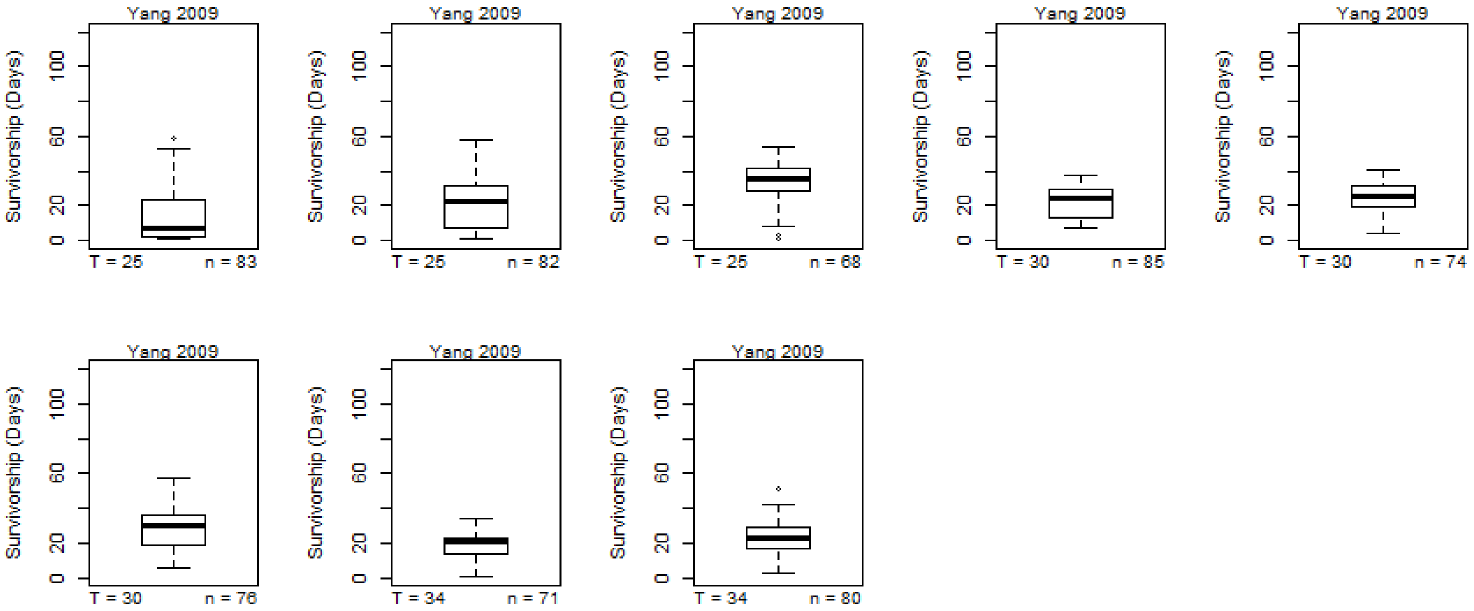


**Figure SI1.** Boxplots of the *Ae. aegypti* survival under controlled conditions in the laboratory for each experiment used in this study, T = the temperature at which the experiment took place, n = the total number of mosquitoes under observation.

## *Ae. aegypti* laboratory experiment bibliography


1. Bargielowski I, Nimmo D, Alphey L, Koella JC: **Comparison of life history characteristics of the genetically modified OX513A line and a wild type strain of *Aedes aegypti***. *PLoS One* 2011, **6**(6):e20699.
2. Briegel H, Knusel I, Timmermann SE: ***Aedes aegypti*: size, reserves, survival, and flight potential**. *Journal of Vector Ecology* 2001, **26**:21-31.
3. Carrington LB, Armijos MV, Lambrechts L, Scott TW: **Fluctuations at a low mean temperature accelerate dengue virus transmission by *Aedes aegypti***. *Plos Neglected Tropical Diseases* 2013, **7**(4):e2190.
4. Crovello TJ, Hacker CS: **Evolutionary strategies in life table characteristics among feral and urban strains of *Aedes aegypti* (L.)**. *Evolution* 1972:185-196.
5. Grech MG, Ludueña Almeida F, Almirón WR: **Bionomics of *Aedes aegypti* subpopulations (Diptera: Culicidae) from Argentina**. *Journal of Vector Ecology* 2010, **35**(2):277-285.
6. Joy TK, Arik AJ, Corby-Harris V, Johnson AA, Riehle MA: **The impact of larval and adult dietary restriction on lifespan, reproduction and growth in the mosquito *Aedes aegypti***. *Experimental gerontology* 2010, **45**(9):685-690.
7. Lambrechts L, Paaijmans KP, Fansiri T, Carrington LB, Kramer LD, Thomas MB, Scott TW: **Impact of daily temperature fluctuations on dengue virus transmission by *Aedes aegypti***. *Proceedings of the National Academy of Sciences* 2011, **108**(18):7460-7465.
8. Lansdowne C, Hacker CS: **The effect of fluctuating temperature and humidity on the adult life table characteristics of five strains of *Aedes aegypti***. *Journal of Medical Entomology* 1975, **11**(6):723-733.

9. Maciel-de-Freitas R, Codeco C, Lourenco de Oliveira R: **Body size associated survival and dispersal rates of *Aedes aegypti* in Rio de Janeiro**. *Medical and Veterinary Entomology* 2007, **21**(3):284-292.
10. McMeniman CJ, Lane RV, Cass BN, Fong AW, Sidhu M, Wang Y-F, O'Neill SL: **Stable introduction of a life-shortening Wolbachia infection into the mosquito *Aedes aegypti***. *Science* 2009, **323**(5910):141-144.
11. Muir LE, Kay BH: ***Aedes aegypti* survival and dispersal estimated by mark-release-recapture in northern Australia**. *The American Journal of Tropical Medicine and Hygiene* 1998, **58**(3):277-282.
12. Reiskind MH, Westbrook CJ, Lounibos LP: **Exposure to chikungunya virus and adult longevity in *Aedes aegypti* (L.) and *Aedes albopictus* (Skuse)**. *Journal of Vector Ecology* 2010, **35**(1):61-68.
13. Richie JP, Mills BJ, Lang CA: **Dietary nordihydroguaiaretic acid increases the life span of the mosquito**. In: *Proceedings of the Society for Experimental Biology and Medicine: 1986; New York City, NY*: Royal Society of Medicine; 1986: 81-85.
14. Richie JP, Mills BJ, Lang CA: **Correction of a glutathione deficiency in the aging mosquito increases its longevity**. In: *Proceedings of the Society for Experimental Biology and Medicine: 1987; New York City, NY*: Royal Society of Medicine; 1987: 113-117.
15. Sawby R, Klowden M, Sjogren R: **Sublethal effects of larval methoprene exposure on adult mosquito longevity**. *Journal of the American Mosquito Control Association* 1992, **8**(3):290.
16. Sheppard P, Macdonald W, Tonn R, Grab B: **The dynamics of an adult population of *Aedes aegypti* in relation to dengue haemorrhagic fever in Bangkok**. *The journal of animal ecology* 1969:661-702.
17. Styer LM, Carey JR, Wang J-L, Scott TW: **Mosquitoes do senesce: departure from the paradigm of constant mortality**. *The American Journal of Tropical Medicine and Hygiene* 2007, **76**(1):111.
18. Sylvestre G, Gandini M, Maciel-de-Freitas R: **Age-dependent effects of oral infection with dengue virus on *Aedes aegypti* (Diptera: Culicidae) feeding behavior, survival, oviposition success and fecundity**. *PLoS One* 2013, **8**(3):e59933.
19. Yang H, Macoris M, Galvani K, Andrighetti M, Wanderley D: **Assessing the effects of temperature on the population of *Aedes aegypti*, the vector of dengue**. *Epidemiology and Infection* 2009, **137**(8):1188.

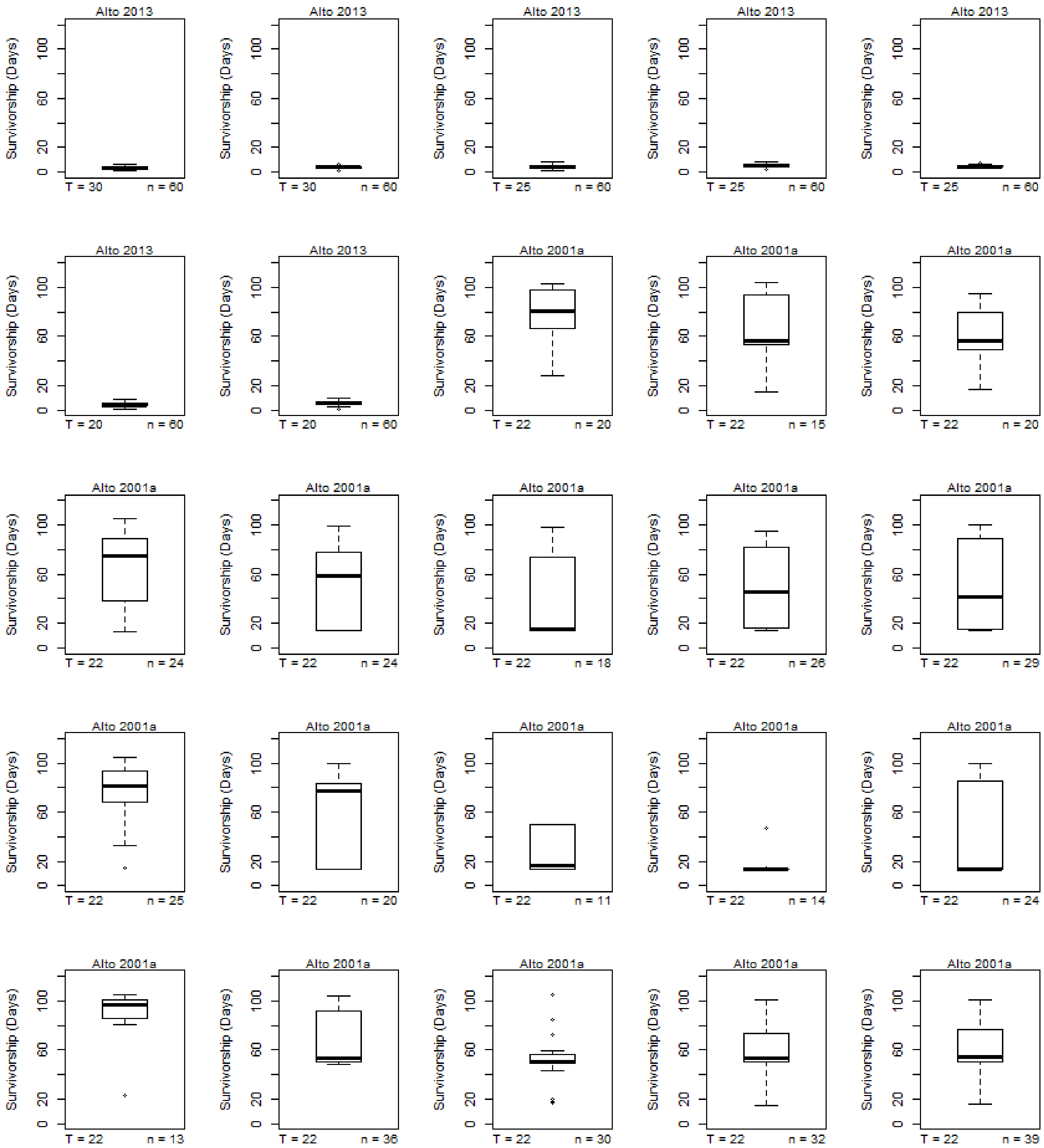
Alto 2013
Survivorship (Days)
0 20 60 100
T = 30 n = 60
Alto 2013
Survivorship (Days)
0 20 60 100
T = 30 n = 60
Alto 2013
Survivorship (Days)
0 20 60 100
T = 25 n = 60
Alto 2013
Survivorship (Days)
0 20 60 100
T = 25 n = 60
Alto 2013
Survivorship (Days)
0 20 60 100
T = 25 n = 60
Alto 2013
Survivorship (Days)
0 20 60 100
T = 20 n = 60
Alto 2013
Survivorship (Days)
0 20 60 100
T = 20 n = 60
Alto 2001a
Survivorship (Days)
0 20 60 100
T = 22 n = 20
Alto 2001a
Survivorship (Days)
0 20 60 100
T = 22 n = 15
Alto 2001a
Survivorship (Days)
0 20 60 100
T = 22 n = 20
Alto 2001a
Survivorship (Days)
0 20 60 100
T = 22 n = 24
Alto 2001a
Survivorship (Days)
0 20 60 100
T = 22 n = 24
Alto 2001a
Survivorship (Days)
0 20 60 100
T = 22 n = 18
Alto 2001a
Survivorship (Days)
0 20 60 100
T = 22 n = 26
Alto 2001a
Survivorship (Days)
0 20 60 100
T = 22 n = 29
Alto 2001a
Survivorship (Days)
0 20 60 100
T = 22 n = 25
Alto 2001a
Survivorship (Days)
0 20 60 100
T = 22 n = 20
Alto 2001a
Survivorship (Days)
0 20 60 100
T = 22 n = 11
Alto 2001a
Survivorship (Days)
0 20 60 100
T = 22 n = 14
Alto 2001a
Survivorship (Days)
0 20 60 100
T = 22 n = 24
Alto 2001a
Survivorship (Days)
0 20 60 100
T = 22 n = 13
Alto 2001a
Survivorship (Days)
0 20 60 100
T = 22 n = 36
Alto 2001a
Survivorship (Days)
0 20 60 100
T = 22 n = 30
Alto 2001a
Survivorship (Days)
0 20 60 100
T = 22 n = 32
Alto 2001a
Survivorship (Days)
0 20 60 100
T = 22 n = 39

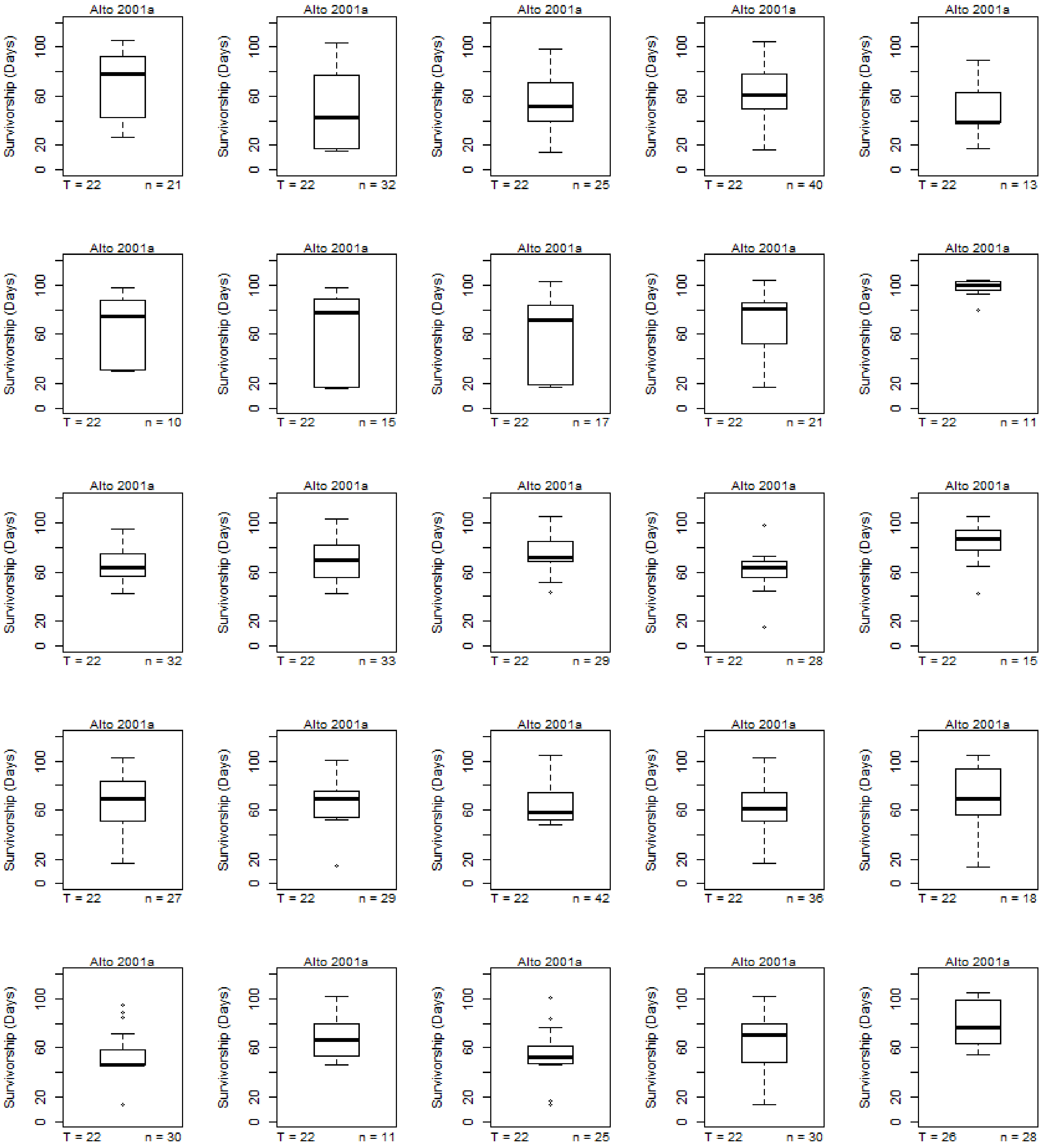

Alto 2001a
Survivorship (Days)
0
20
60
100
T = 22
n = 21
n = 32
n = 25
n = 40
n = 13
n = 10
n = 15
n = 17
n = 21
n = 11
n = 32
n = 33
n = 29
n = 28
n = 15
n = 27
n = 29
n = 42
n = 36
n = 18
n = 30
n = 11
n = 25
n = 30
T = 26
n = 28

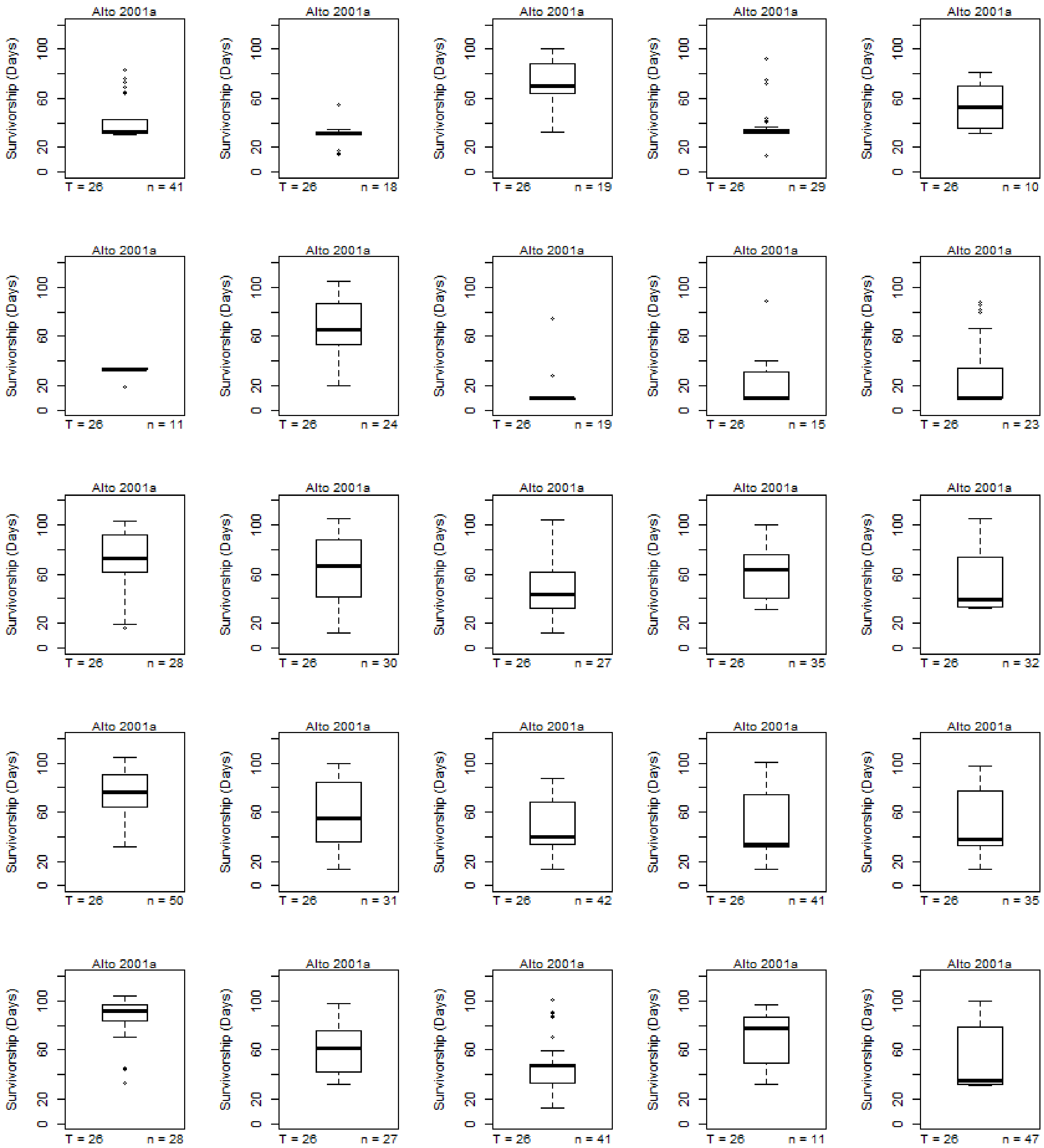

Alto 2001a
Survivorship (Days)
0
20
60
100
T = 26
n = 41
n = 18
n = 19
n = 29
n = 10
n = 11
n = 24
n = 19
n = 15
n = 23
n = 28
n = 30
n = 27
n = 35
n = 32
n = 50
n = 31
n = 42
n = 41
n = 35
n = 28
n = 27
n = 41
n = 11
n = 47

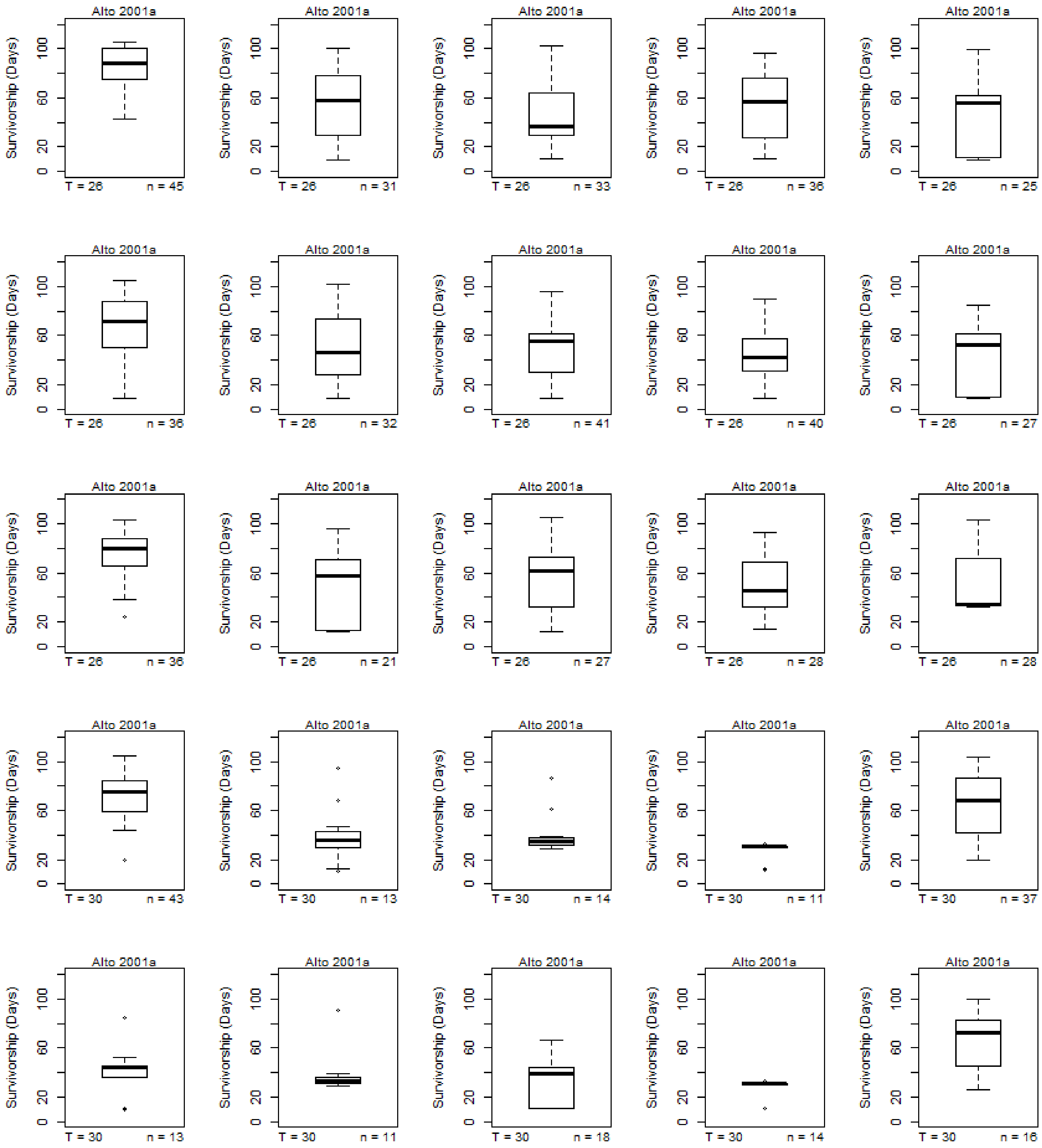
Alto 2001a
Survivorship (Days)
0 20 60 100
T = 26
n = 45
Alto 2001a
Survivorship (Days)
0 20 60 100
T = 26
n = 31
Alto 2001a
Survivorship (Days)
0 20 60 100
T = 26
n = 33
Alto 2001a
Survivorship (Days)
0 20 60 100
T = 26
n = 36
Alto 2001a
Survivorship (Days)
0 20 60 100
T = 26
n = 25
Alto 2001a
Survivorship (Days)
0 20 60 100
T = 26
n = 36
Alto 2001a
Survivorship (Days)
0 20 60 100
T = 26
n = 32
Alto 2001a
Survivorship (Days)
0 20 60 100
T = 26
n = 41
Alto 2001a
Survivorship (Days)
0 20 60 100
T = 26
n = 40
Alto 2001a
Survivorship (Days)
0 20 60 100
T = 26
n = 27
Alto 2001a
Survivorship (Days)
0 20 60 100
T = 26
n = 36
Alto 2001a
Survivorship (Days)
0 20 60 100
T = 26
n = 21
Alto 2001a
Survivorship (Days)
0 20 60 100
T = 26
n = 27
Alto 2001a
Survivorship (Days)
0 20 60 100
T = 26
n = 28
Alto 2001a
Survivorship (Days)
0 20 60 100
T = 26
n = 28
Alto 2001a
Survivorship (Days)
0 20 60 100
T = 30
n = 43
Alto 2001a
Survivorship (Days)
0 20 60 100
T = 30
n = 13
Alto 2001a
Survivorship (Days)
0 20 60 100
T = 30
n = 14
Alto 2001a
Survivorship (Days)
0 20 60 100
T = 30
n = 11
Alto 2001a
Survivorship (Days)
0 20 60 100
T = 30
n = 37
Alto 2001a
Survivorship (Days)
0 20 60 100
T = 30
n = 13
Alto 2001a
Survivorship (Days)
0 20 60 100
T = 30
n = 11
Alto 2001a
Survivorship (Days)
0 20 60 100
T = 30
n = 18
Alto 2001a
Survivorship (Days)
0 20 60 100
T = 30
n = 14
Alto 2001a
Survivorship (Days)
0 20 60 100
T = 30
n = 16

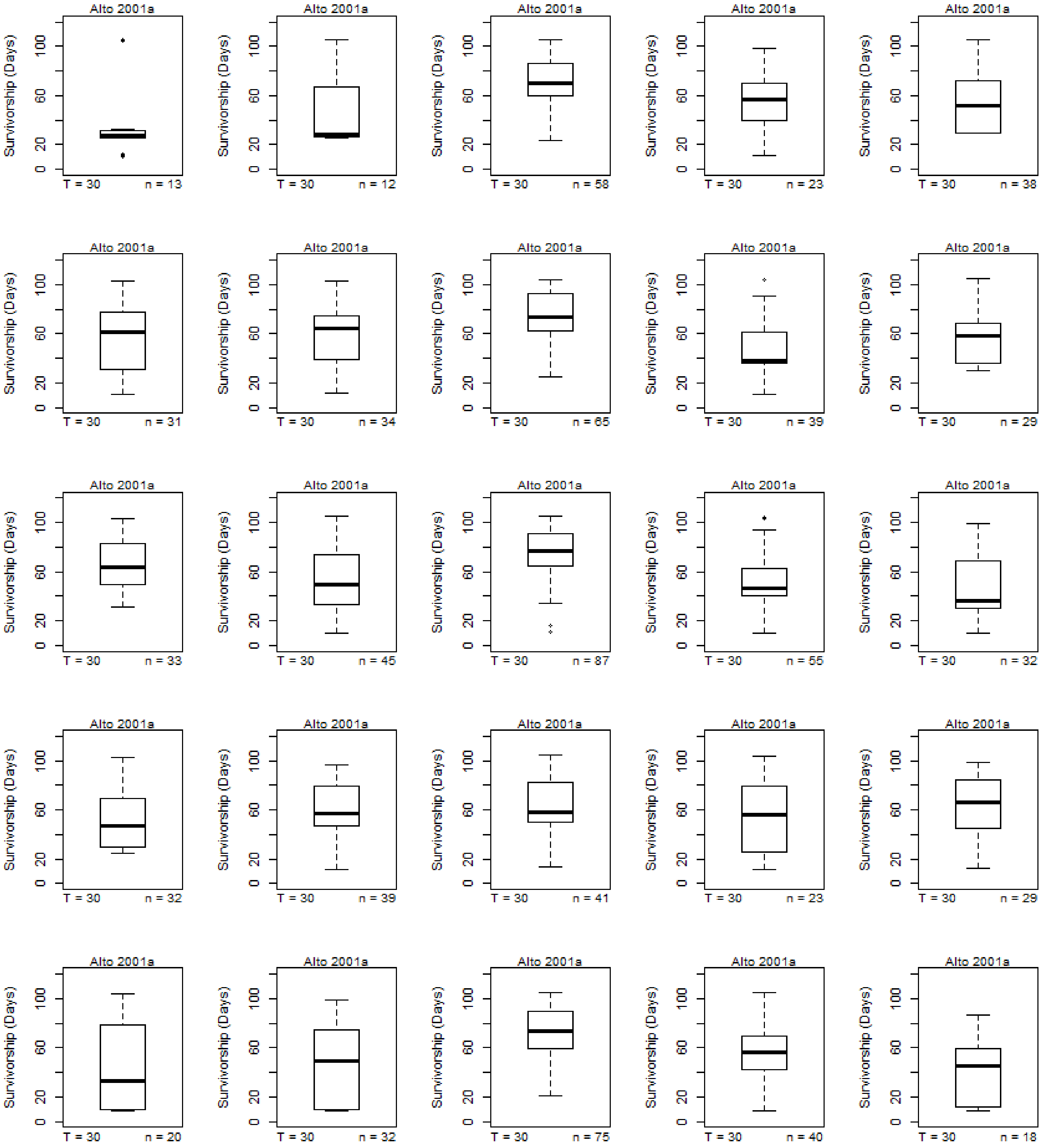
Alto 2001a
Survivorship (Days)
0
20
60
100
T = 30
n = 13
Alto 2001a
T = 30
n = 12
Alto 2001a
T = 30
n = 58
Alto 2001a
T = 30
n = 23
Alto 2001a
T = 30
n = 38
Alto 2001a
T = 30
n = 31
Alto 2001a
T = 30
n = 34
Alto 2001a
T = 30
n = 65
Alto 2001a
T = 30
n = 39
Alto 2001a
T = 30
n = 29
Alto 2001a
T = 30
n = 33
Alto 2001a
T = 30
n = 45
Alto 2001a
T = 30
n = 87
Alto 2001a
T = 30
n = 55
Alto 2001a
T = 30
n = 32
Alto 2001a
T = 30
n = 32
Alto 2001a
T = 30
n = 39
Alto 2001a
T = 30
n = 41
Alto 2001a
T = 30
n = 23
Alto 2001a
T = 30
n = 29
Alto 2001a
T = 30
n = 20
Alto 2001a
T = 30
n = 32
Alto 2001a
T = 30
n = 75
Alto 2001a
T = 30
n = 40
Alto 2001a
T = 30
n = 18

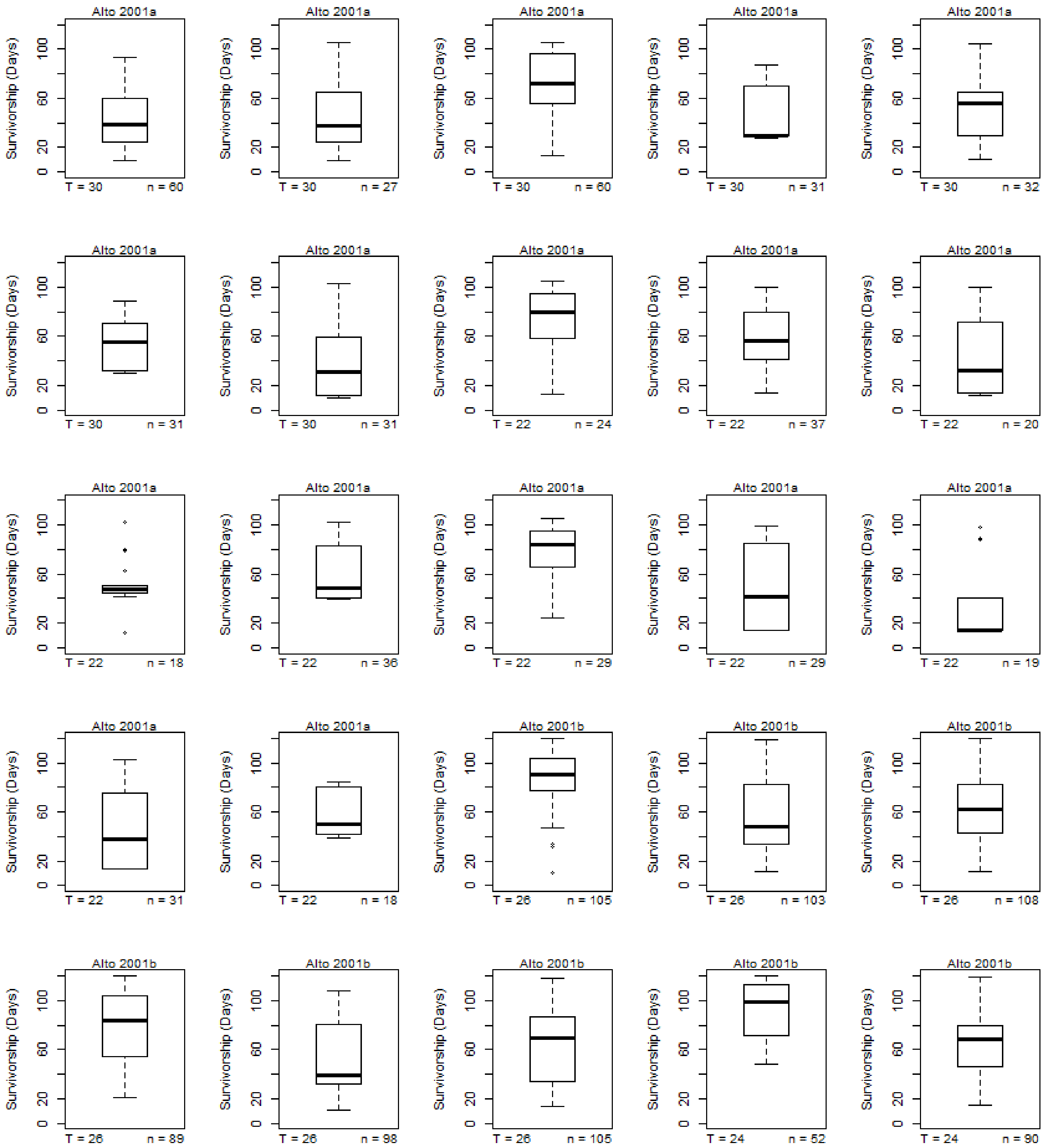

Alto 2001a
Survivorship (Days)
0 20 60 100
T = 30 n = 60
Alto 2001a
T = 30 n = 27
Alto 2001a
T = 30 n = 60
Alto 2001a
T = 30 n = 31
Alto 2001a
T = 30 n = 32
Alto 2001a
T = 30 n = 31
Alto 2001a
T = 30 n = 31
Alto 2001a
T = 22 n = 24
Alto 2001a
T = 22 n = 37
Alto 2001a
T = 22 n = 20
Alto 2001a
T = 22 n = 18
Alto 2001a
T = 22 n = 36
Alto 2001a
T = 22 n = 29
Alto 2001a
T = 22 n = 29
Alto 2001a
T = 22 n = 19
Alto 2001a
T = 22 n = 31
Alto 2001a
T = 22 n = 18
Alto 2001b
T = 26 n = 105
Alto 2001b
T = 26 n = 103
Alto 2001b
T = 26 n = 108
Alto 2001b
T = 26 n = 89
Alto 2001b
T = 26 n = 98
Alto 2001b
T = 26 n = 105
Alto 2001b
T = 24 n = 52
Alto 2001b
T = 24 n = 90

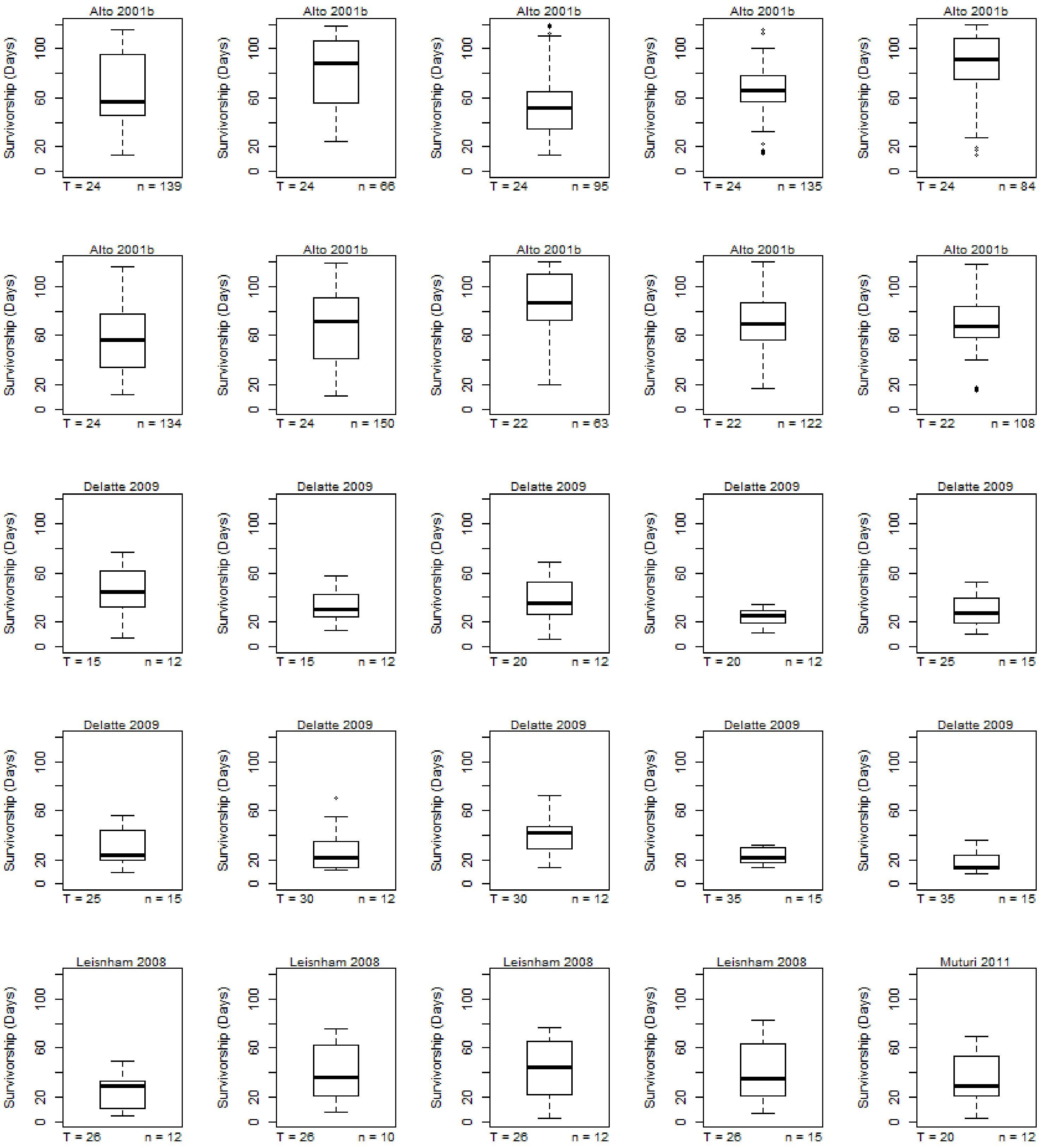

Alto 2001b
Survivorship (Days)
0 20 60 100
T = 24 n = 139
Alto 2001b
T = 24 n = 66
Alto 2001b
T = 24 n = 95
Alto 2001b
T = 24 n = 135
Alto 2001b
T = 24 n = 84
Alto 2001b
T = 24 n = 134
Alto 2001b
T = 24 n = 150
Alto 2001b
T = 22 n = 63
Alto 2001b
T = 22 n = 122
Alto 2001b
T = 22 n = 108
Delatte 2009
T = 15 n = 12
Delatte 2009
T = 15 n = 12
Delatte 2009
T = 20 n = 12
Delatte 2009
T = 20 n = 12
Delatte 2009
T = 25 n = 15
Delatte 2009
T = 25 n = 15
Delatte 2009
T = 30 n = 12
Delatte 2009
T = 30 n = 12
Delatte 2009
T = 35 n = 15
Delatte 2009
T = 35 n = 15
Leisnham 2008
T = 26 n = 12
Leisnham 2008
T = 26 n = 10
Leisnham 2008
T = 26 n = 12
Leisnham 2008
T = 26 n = 15
Muturi 2011
T = 20 n = 12

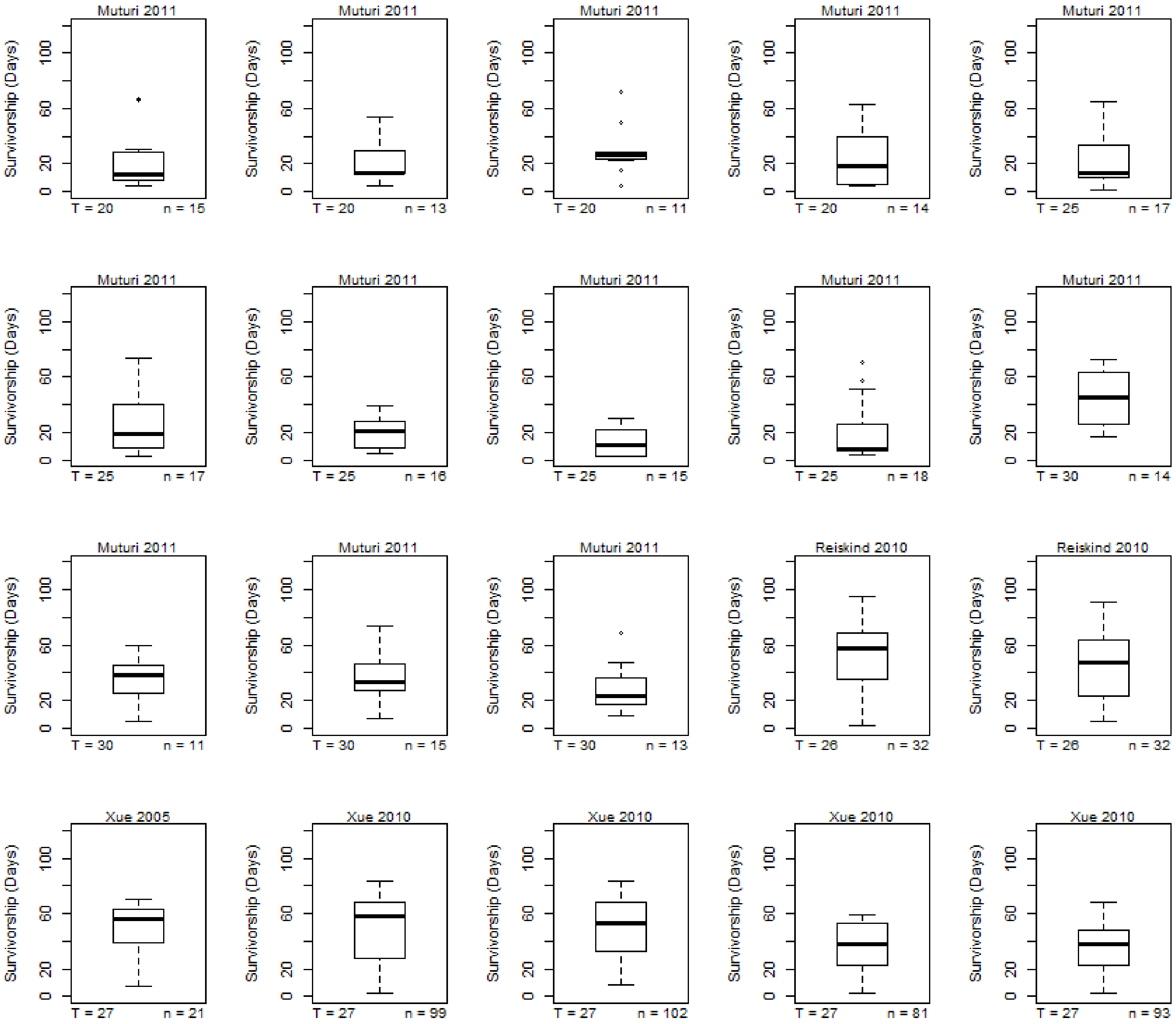


**Figure SI2.** Boxplots of *Ae. albopictus* survival under controlled conditions in the laboratory for each experiment used in this study, T = the temperature at which the experiment took place, n = the total number of mosquitoes under observation.

## *Ae. albopictus* laboratory experiment bibliography

1. Alto BW, Bettinardi D: **Temperature and dengue virus infection in mosquitoes: independent effects on the immature and adult stages**. *The American Journal of Tropical Medicine and Hygiene* 2013, **88**(3):497-505.

2. Alto BW, Juliano SA: **Precipitation and temperature effects on populations of *Aedes albopictus* (Diptera: Culicidae): implications for range expansion**. *Journal of Medical Entomology* 2001, **38**(5):646.
3. Alto BW, Juliano SA: **Temperature effects on the dynamics of *Aedes albopictus* (Diptera: Culicidae) populations in the laboratory**. *Journal of Medical Entomology* 2001, **38**(4):548.
4. Delatte H, Gimonneau G, Triboire A, Fontenille D: **Influence of temperature on immature development, survival, longevity, fecundity, and gonotrophic cycles of *Aedes albopictus*, vector of chikungunya and dengue in the Indian Ocean**. *Journal of Medical Entomology* 2009, **46**(1):33-41.
5. Leisnham P, Sala L, Juliano S: **Geographic variation in adult survival and reproductive tactics of the mosquito *Aedes albopictus***. *Journal of Medical Entomology* 2008, **45**(2):210.
6. Muturi EJ, Lampman R, Costanzo K, Alto BW: **Effect of temperature and insecticide stress on life-history traits of *Culex* restuans and *Aedes albopictus* (Diptera: Culicidae)**. *Journal of Medical Entomology* 2011, **48**(2):243-250.
7. Reiskind MH, Westbrook CJ, Lounibos LP: **Exposure to chikungunya virus and adult longevity in *Aedes aegypti* (L.) and *Aedes albopictus* (Skuse)**. *Journal of Vector Ecology* 2010, **35**(1):61-68.
8. Xue R, Ali A, Barnard D: **Effects of forced egg-retention in *Aedes albopictus* on adult survival and reproduction following application of DEET as an oviposition deterrent**. *Journal of Vector Ecology* 2005, **30**(1):45.
9. Xue R-D, Barnard DR, Muller GC: **Effects of body size and nutritional regimen on survival in adult *Aedes albopictus* (Diptera: Culicidae)**. *Journal of Medical Entomology* 2010, **47**(5):778-782.

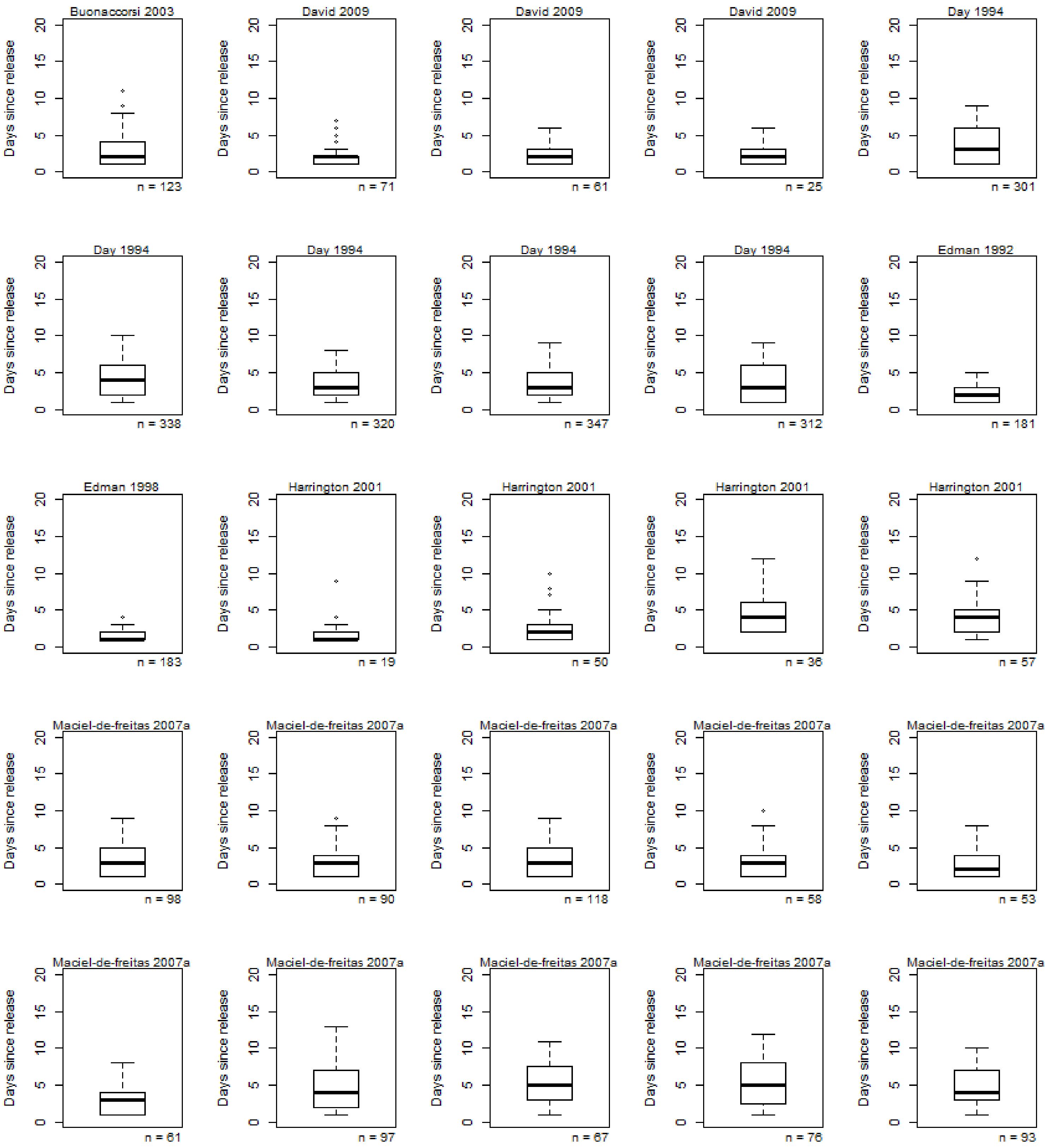
Days since release
0 5 10 15 20
Buonaccorsi 2003
n = 123
David 2009
n = 71
David 2009
n = 61
David 2009
n = 25
Day 1994
n = 301
Day 1994
n = 338
Day 1994
n = 320
Day 1994
n = 347
Day 1994
n = 312
Edman 1992
n = 181
Edman 1998
n = 183
Harrington 2001
n = 19
Harrington 2001
n = 50
Harrington 2001
n = 36
Harrington 2001
n = 57
Maciel-de-freitas 2007a
n = 98
Maciel-de-freitas 2007a
n = 90
Maciel-de-freitas 2007a
n = 118
Maciel-de-freitas 2007a
n = 58
Maciel-de-freitas 2007a
n = 53
Maciel-de-freitas 2007a
n = 61
Maciel-de-freitas 2007a
n = 97
Maciel-de-freitas 2007a
n = 67
Maciel-de-freitas 2007a
n = 76
Maciel-de-freitas 2007a
n = 93

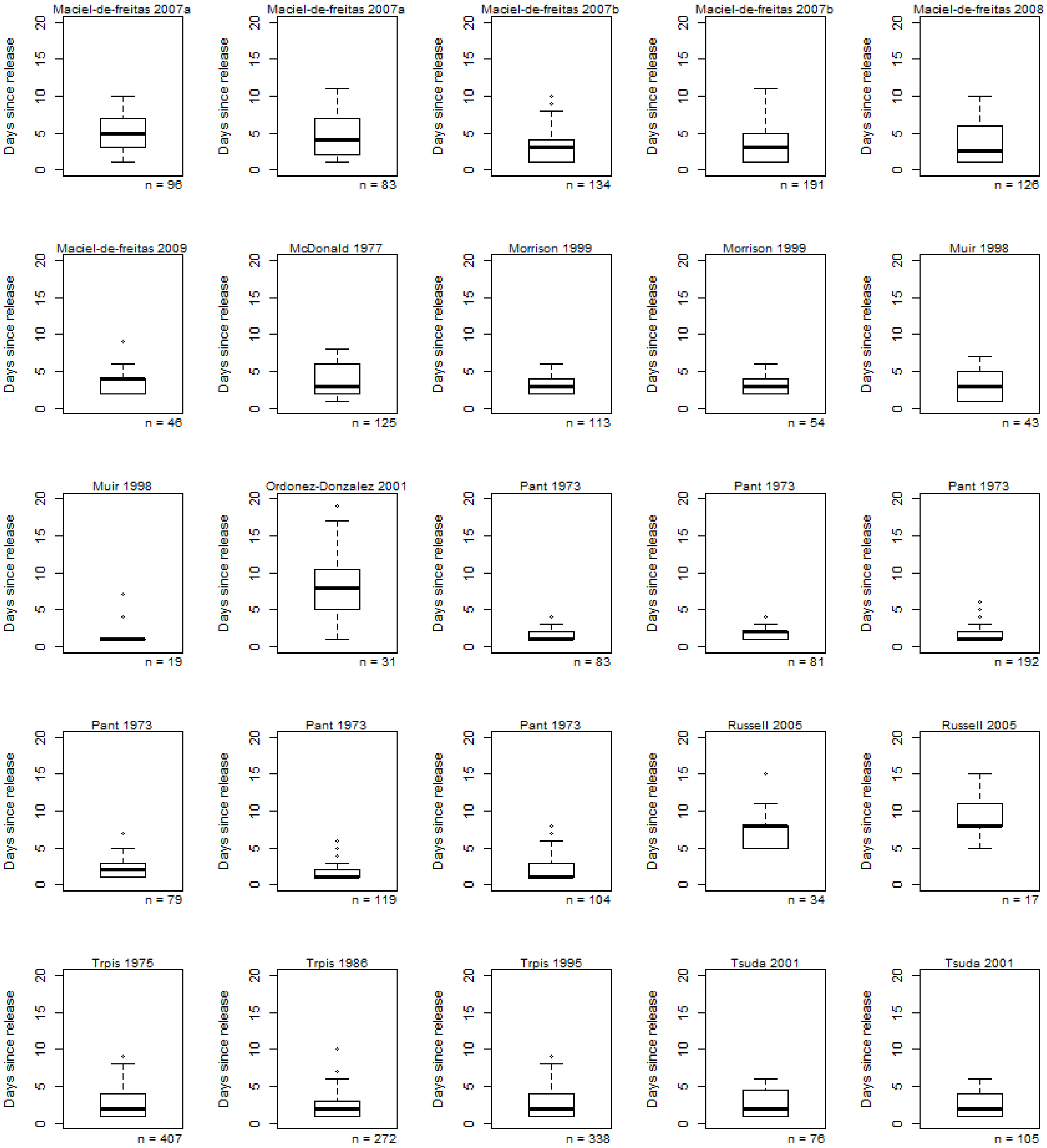


**Figure SI3.** Boxplots of the proportions of *Ae. aegypti* recaptures on successive days post release for each experiment used in this study, n = the total number of mosquitoes recaptured.

# *Ae. aegypti* mark-release-recapture experiment bibliography


1. Buonaccorsi JP, Harrington LC, Edman JD: **Estimation and comparison of mosquito survival rates with release-recapture-removal data**. *Journal of Medical Entomology* 2003, **40**(1):6-17.
2. David MR, Lourenço-de-Oliveira R, Freitas RMd: **Container productivity, daily survival rates and dispersal of *Aedes aegypti* mosquitoes in a high income dengue epidemic neighbourhood of Rio de Janeiro: presumed influence of differential urban structure on mosquito biology**. *Memorias Do Instituto Oswaldo Cruz* 2009, **104**(6):927-932.
3. Day JF, Edman JD, Scott TW: **Reproductive fitness and survivorship of *Aedes aegypti* (Diptera: Culicidae) maintained on blood, with field observations from Thailand**. *Journal of Medical Entomology* 1994, **31**(4):611-617.
4. Edman JD, Strickman D, Kittayapong P, Scott TW: **Female *Aedes aegypti* (Diptera: Culicidae) in Thailand rarely feed on sugar**. *Journal of Medical Entomology* 1992, **29**(6):1035-1038.
5. Edman JD, Scott TW, Costero A, Morrison AC, Harrington LC, Clark GG: ***Aedes aegypti* (Diptera: Culicidae) movement influenced by availability of oviposition sites**. *Journal of Medical Entomology* 1998, **35**(4):578-583.
6. Harrington LC, Buonaccorsi JP, Edman JD, Costero A, Kittayapong P, Clark GG, Scott TW: **Analysis of survival of young and old *Aedes aegypti* (Diptera: Culicidac) from Puerto Rico and Thailand**. *Journal of Medical Entomology* 2001, **38**(4):537-547.
7. Maciel-de-Freitas R, Codeco CT, Lourenco-de-Oliveira R: **Daily survival rates and dispersal of *Aedes aegypti* females in Rio de Janeiro, Brazil**. *Am J Trop Med Hyg* 2007, **76**(4):659-665.
8. Maciel-de-Freitas R, Codeco C, Lourenco de Oliveira R: **Body size associated survival and dispersal rates of *Aedes aegypti* in Rio de Janeiro**. *Medical and Veterinary Entomology* 2007, **21**(3):284-292.
9. Maciel-de-Freitas R, Eiras AE, Lourenco-de-Oliveira R: **Calculating the survival rate and estimated population density of gravid *Aedes aegypti* (Diptera, Culicidae) in Rio de Janeiro, Brazil**. *Cad Saude Publica* 2008, **24**(12):2747-2754.
10. Maciel-de-Freitas R, Lourenco-de-Oliveira R: **Presumed unconstrained dispersal of *Aedes aegypti* in the city of Rio de Janeiro, Brazil**. *Rev Saude Publica* 2009, **43**(1):8-12.
11. McDonald PT: **Population characteristics of domestic *Aedes aegypti* (Diptera: Culicidae) in villages on the Kenya Coast I. Adult survivorship and population size**. *Journal of Medical Entomology* 1977, **14**(1):42-48.
12. Morrison AC, Costero A, Edman JD, Clark GG, Scott TW: **Increased fecundity of *Aedes aegypti* fed human blood before release in a mark-recapture study in Puerto Rico**. *Journal of the American Mosquito Control Association* 1999, **15**(2):98-104.
13. Muir LE, Kay BH: ***Aedes aegypti* survival and dispersal estimated by mark-release-recapture in northern Australia**. *The American Journal of Tropical Medicine and Hygiene* 1998, **58**(3):277-282.
14. Ordonez-Gonzalez JG, Mercado-Hernandez R, Flores-Suarez AE, Fernandez-Salas I: **The use of sticky ovitraps to estimate dispersal of *Aedes aegypti* in Northeastern Mexico**. *Journal of the American Mosquito Control Association* 2001, **17**(2):93-97.
15. Pant CP, Yasuno M: **Field studies on the gonotrophic cycle of *Aedes aegypti* in Bangkok, Thailand**. *Journal of Medical Entomology* 1973, **10**(2):219-223.
16. Russell RC, Webb CE, Williams CR, Ritchie SA: **Mark-release-recapture study to measure dispersal of the mosquito *Aedes aegypti* in Cairns, Queensland, Australia**. *Medical and Veterinary Entomology* 2005, **19**(4):451-457.

17. Trpis M, Hausermann W: **Demonstration of differential domesticity of *Aedes aegypti* (L.)(Diptera, Culicidae) in Africa by mark-release-recapture**. *Bulletin of Entomological Research* 1975, **65**(02):199-208.
18. Trpis M, Hausermann W: **Dispersal and other population parameters of *Aedes aegypti* in an African village and their possible significance in epidemiology of vector-borne diseases**. *Am J Trop Med Hyg* 1986, **35**(6):1263-1279.
19. Trpis M, Hausermann W, Craig GB, Jr.: **Estimates of population size, dispersal, and longevity of domestic *Aedes aegypti* aegypti (Diptera: Culicidae) by mark-release-recapture in the village of Shauri Moyo in eastern Kenya**. *Journal of Medical Entomology* 1995, **32**(1):27-33.
20. Tsuda Y, Takagi M, Wang S, Wang Z, Tang L: **Movement of *Aedes aegypti* (Diptera: Culicidae) released in a small isolated village on Hainan Island, China**. *Journal of Medical Entomology* 2001, **38**(1):93-98.

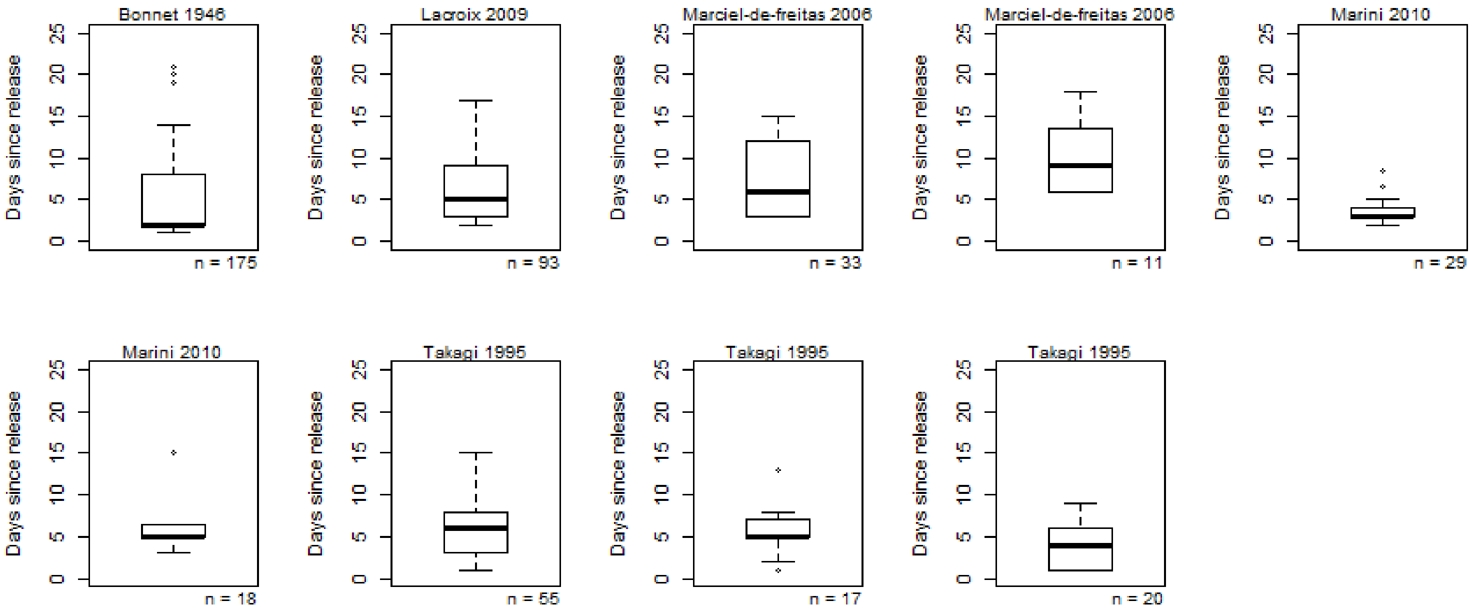


**Figure SI4.** Boxplots of the proportions of *Ae. albopictus* recaptures on successive days post release for each experiment used in this study, n = the total number of mosquitoes recaptured.

## *Ae. albopictus* mark-release-recapture experiment bibliography


1. Bonnet DD, Worcester DJ: **The dispersal of *Aedes albopictus* in the territory of Hawaii**. *The American Journal of Tropical Medicine and Hygiene* 1946, **1**(4):465.
2. Lacroix R, Delatte H, Hue T, Reiter P: **Dispersal and survival of male and female *Aedes albopictus* (Diptera: Culicidae) on Reunion Island**. *Journal of Medical Entomology* 2009, **46**(5):1117-1124.
3. Maciel de Freitas R, Neto RB, Goncalves JM, Codeco CT, Lourenco-de-Oliveira R: **Movement of dengue vectors between the human modified environment and an urban forest in Rio de Janeiro**. *Journal of Medical Entomology* 2006, **43**(6):1112-1120.
4. Marini F, Caputo B, Pombi M, Tarsitani G, della Torre A: **Study of *Aedes albopictus* dispersal in Rome, Italy, using sticky traps in mark-release-recapture experiments**. *Medical and Veterinary Entomology* 2010, **24**(4):361-368.
5. Takagi M, Tsuda Y, Suzuki A, Wada Y: **Movement of individually marked *Aedes albopictus* females in Nagasaki, Japan**. *Trop Med* 1995, **37**(2):79-85.
